# Genetically Predicted Vegetable Intake and Cardiovascular Diseases and Risk Factors: An Investigation with Mendelian Randomization

**DOI:** 10.3390/nu15173682

**Published:** 2023-08-22

**Authors:** Qi Feng, Andrew J. Grant, Qian Yang, Stephen Burgess, Jelena Bešević, Megan Conroy, Wemimo Omiyale, Yangbo Sun, Naomi Allen, Ben Lacey

**Affiliations:** 1 Nuffield Department of Population Health, University of Oxford, Oxford OX3 7LF, UK; 2 MRC Biostatistics Unit, University of Cambridge, Cambridge CB2 0SZ, UK; 3 MRC Integrative Epidemiology, University of Bristol, Bristol BS1 3NY, UK; 4 Population Health Sciences, Bristol Medical School, University of Bristol, Bristol BS1 3NY, UK; 5 Department of Preventive Medicine, The University of Tennessee Health Science Center, Memphis, TN 38163, USA

**Keywords:** vegetable intake, raw vegetable, cooked vegetable, Mendelian randomization, UK Biobank, polygenic risk score, cardiovascular diseases, cardiometabolic risk factors

## Abstract

Background: The associations between vegetable intake and cardiovascular diseases have been demonstrated in observational studies, but less sufficiently in randomized trials. Mendelian randomization has been considered a promising alternative in causal inference. The separate effects of cooked and raw vegetable intake remain unclear. This study aimed to investigate the associations between cooked and raw vegetable intake with cardiovascular outcomes using MR. Methods: We identified 15 and 28 genetic variants statistically and biologically associated with cooked and raw vegetable intake, respectively, from previous genome-wide association studies, which were used as instrumental variables to estimate associations with coronary heart disease (CHD), stroke, heart failure (HF), and atrial fibrillation (AF). The independent effects of genetically predicted cooked and raw vegetable intake were examined using multivariable MR analysis. We performed one-sample and two-sample MR analyses and combined their results using meta-analysis. Bonferroni correction was applied for multiple comparisons. We performed two-sample MR analysis for cardiometabolic risk factors (serum lipids, blood pressure, body mass index, and glycemic traits) to explore the potential mechanisms. Results: In the MR meta-analysis of 1.2 million participants, we found null evidence for associations between genetically predicted cooked and raw vegetable intake with CHD, HF, or AF. Raw vegetable intake was nominally associated with stroke (odds ratio [95% confidence interval] 0.82 [0.69–0.98] per 1 daily serving increase, *p* = 0.03), but this association did not pass the corrected significance level. We found consistently null evidence for associations with serum lipids, blood pressure, body mass index, or glycemic traits. Conclusions: We found null evidence for associations between genetically predicted vegetable intake with CHD, AF, HF, or cardiometabolic risk factors in this MR study. Raw vegetable intake may reduce risk of stroke, but this warrants more research. True associations between vegetable intake and CVDs cannot be completely ruled out, and future investigations are required for causal inference in nutritional research.

## 1. Introduction

Cardiovascular diseases (CVDs) are the leading cause of global burden of disease [1] and are caused by a complex interplay of genetic and environmental factors [2,3]. It is estimated that 8 million CVD-related deaths and 188 million CVD disability-adjusted life years are attributable to unhealthy diets annually [3], of which up to 20% may be due to insufficient vegetable intake [4].

There exists a substantial body of observational evidence supporting inverse associations between vegetable intake and cardiovascular diseases and risk factors [5,6], which has led to international dietary guidelines recommending higher intake of vegetables for primary prevention [7,8,9]. A meta-analysis of 45 cohort studies found that higher vegetable intake was associated with a 13% lower risk of CVDs [10], with other meta-analyses reporting similar risk reductions in coronary heart disease (CHD) and stroke [11]. In spite of large sample sizes, long follow-up periods, and adjustment for multiple confounders in some studies [12,13,14], observational associations have been criticized for predisposition to residual confounding. The residual confounding may result from unmeasured covariates and/or imperfect measurement of adjusted confounders, because vegetable intake tends to be correlated with socioeconomic status and lifestyle, among other factors, which are difficult to measure accurately [15]. A previous study suggested that residual confounding accounted for a large proportion of the observed association [16].

So far, evidence from randomized controlled trials has been limited. For example, a meta-analysis of eight trials, including a total of 400 individuals, demonstrated null effects of increasing vegetable intake on systolic blood pressure (SBP), fasting glucose (FG), high-density lipoprotein (HDL), or triglyceride [17]. Another meta-analyses of 12 trials, including 1000 individuals, reported a small reduction in body weight (0.68 kg) following a 14-week isocaloric diet with high vegetable content [6]. These trials suggest limited evidence for the effects of increasing vegetable intake on cardiometabolic risk factors, in contrast to observational evidence, which could be due to short periods of intervention and follow up. These trials mainly examined intermediate cardiometabolic risk factors, which are indirect evidence regarding the effects on hard clinical outcomes of incident CVDs. In addition, most studies have examined vegetable intake as a whole and the separate effects of raw and cooked vegetable intake remain inconsistent [12,14,16].

Mendelian randomization (MR) is a study design using genetic variants, usually single nucleotide polymorphisms (SNPs), as instrumental variables to uncover causal relationships between modifiable risk factors, intermediate traits, and health outcomes. MR is less inclined to confounding and reverse causation than conventional observational studies because SNPs are randomly allocated at meiosis and fixed after fertilization, and thus cannot be affected by socio-demographic, behavioral factors, or health status, resembling the principles of randomized controlled trials and generating more valid effect estimates [18]. When randomized trial evidence is insufficient, MR has been considered as a promising alternative in causal inference [19]. MR has been widely applied in causal inference for a wide range of risk factors and health outcomes. The objective of this study was to investigate the effects of cooked and raw vegetable intake on CVD risk using an MR approach.

## 2. Methods

**Figure 1:**
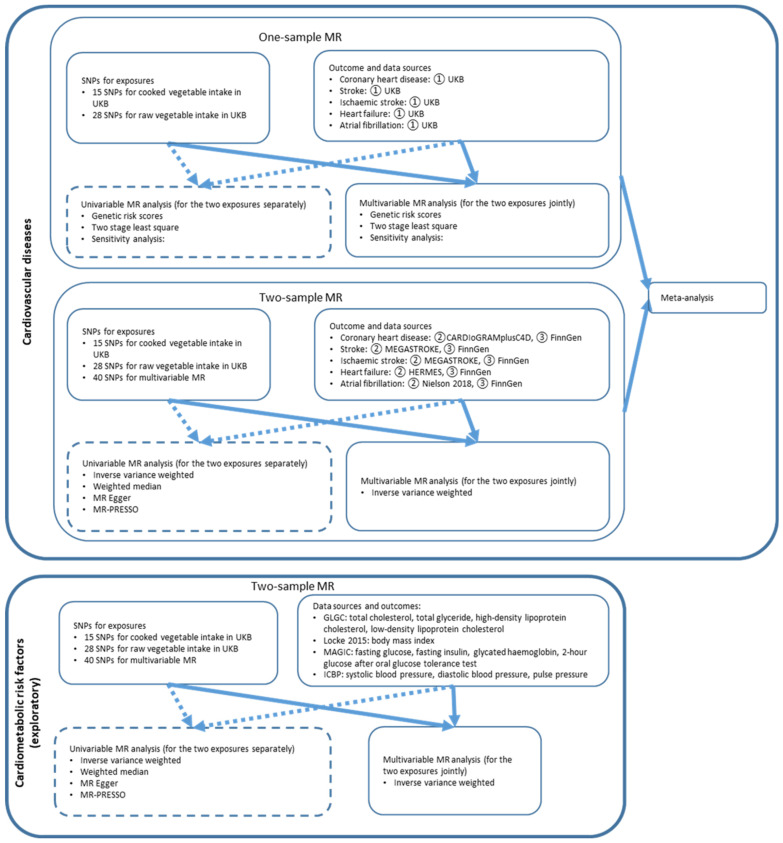
The flowchart of the analysis on genetically predicted vegetable intake with cardiovascular diseases. Solid line: primary analysis. Dashed line: secondary analysis. MR: Mendelian randomization. SNP: single nucleotide polymorphism. UKB: UK Biobank. MR-PRESSO: Mendelian Ran-domization Pleiotropy Residual Sum and Outlier method. CARDIoGRAMplusC4D: Coronary ARtery DIsease Genome-wide Replication and Meta-analysis plus The Coronary Artery Disease Genetics) consortium. MEGASTROKE: MEGASTROKE consortium. HERMES: HEart failure Molecular Epidemiology for therapeutic targetS consortium. GLGC: Global Lipids Genetics Consortium. MAGIC: Meta-Analyses of Glucose and Insulin-related traits Consortium. ICBP: International Consortium for Blood Pressure.

In this study, we performed both two-sample and one-sample MR to quantify the associations between genetically predicted vegetable intake and cardiovascular outcomes. Two-sample MR was performed with summary-level statistics of genome-wide association studies (GWAS), and one-sample MR was performed with individual-level data from the UK Biobank. Two-sample and one-sample MR estimates were meta-analyzed to obtain overall effect estimates. We performed multivariable analysis as the primary analysis, in which cooked and raw vegetable intake was adjusted for the other, aiming to examine their separate effects. Univariable analysis, in which the effects of cooked and raw vegetable intake were fitted separately, was performed as secondary analysis. An overview of the methods is shown in Figure 1.

### 2.1. Genetic Instrument Selection

Genetic instruments associated with cooked and raw vegetable intake were identified in three GWAS from the UK Biobank [20,21,22] (Supplementary Table S1). Individual intake of cooked and raw vegetables (in number of heaped tablespoons; one heaped tablespoon is roughly equivalent to one serving in the UK) was measured using a food frequency questionnaire at recruitment. The repeatability and validity of this questionnaire in the UK Biobank were evaluated and confirmed in a previous analysis: the repeatability was 82% for cooked vegetables and 72% for raw vegetables when compared to a repeat assessment after four years, and high agreement was observed when compared to a 24-hour diet recall assessment [23].

We combined all SNPs that were significant at a genome-wide significance level (*p* < 5 × 10^−8^) in the three GWAS [20,21,22], and removed duplicates, rare variants (minor allele frequency < 1%), or those in linkage disequilibrium (r^2^ > 0.001 or distance > 10,000 kb). To further reduce horizontal pleiotropy, we searched the associated phenotypes for each SNP in the PhenoScanner v2 database (http://www.phenoscanner.medschl.cam.ac.uk/, accessed on 15 July 2023), and further removed SNPs that were associated with potential confounders, such as smoking, alcohol drinking, blood pressure, and adiposity. A similar approach was previously used to identify valid SNPs for vegetable intake in the UK Biobank [24], but our method was able to identify more SNPs by incorporating three relevant GWAS.

In total, we identified 15 and 28 eligible SNPs associated with cooked and raw vegetable intake, which explained 0.8% and 2.4% of phenotypic variance, respectively. The SNPs were located in different gene loci. The majority of the loci were expressed in tissues of the gastrointestinal tract and/or other organs of the digestive system (Supplementary Table S2). The biological mechanisms behind the selected SNPs and vegetable consumption were suggested to be mediated by the individual’s taste and smell preference, as some hit SNPs (for example, *rs9323534 [OR4K17]*) were associated with olfactory receptors [21]. The mechanisms were possibly additionally mediated by their expression in and/or regulation of lipid metabolism (*rs17714824 [EBF1*, *FABP6]*, *rs33947258 [PCDH1]*, *rs12190945 [ME1]*, *rs78940216 [DPYSLS]*, *rs17075255 [MAT2B]*, *rs11608727 [MVK]*) and protein and/or glucose metabolism (*rs6975898 [FOXK1]*, *rs11209780 [NEGR1]*, *rs57221424 [DPY19L2]*, *rs6079589 [MACROD2]*). SNPs associated with raw vegetable intake were specifically associated with gastrointestinal diseases (*rs11125813 [ARMH3]* for Crohn’s disease, *rs4281874 [MVK]* for gastrointestinal dismay), while one SNP associated with cooked vegetable intake was associated with tooth development (*rs10161952 [PHHF2]*). All of these may contribute to an individual’s dietary preference and eventually influence their food consumption (Supplementary Table S2).

The magnitude of the associations between the SNPs and vegetable intake was extracted from the GWAS conducted by Canela-Xandri et al. [22], as this GWAS had a larger sample size and was adjusted for more covariates. It was performed in 452,264 unrelated individuals of European ancestry, and adjusted for sex, age, age square, array batch, assessment center, and the leading 20 genetic principle components. The strength of the genetic instruments was evaluated using the F-statistic, with F-statistic > 10 suggesting good instrument strength [25]. The process of SNP selection and the characteristics of these SNPs are shown in Supplementary Figure S1 and Table S1. 

### 2.2. One-Sample MR

#### 2.2.1. Data Source

We used individual-level data from UK Biobank participants for one-sample MR. The UK Biobank is a population-based prospective cohort that recruited a half-million participants aged 40–69 years between 2006 and 2010 across England, Wales, and Scotland [26]. At baseline, participants completed a touchscreen questionnaire that collected information on socioeconomic status, health status, medication use, lifestyle, and environmental exposures. Anthropometric and physical traits were measured; blood, urine, and saliva samples were collected [26]. Genotypes in the UK Biobank were assayed using the Affymetrix UKBiLEVE Axiom array^®^ for about 50,000 participants and the UK Biobank Axiom array^®^ for about 440,000 participants. Genetic pre-imputation quality control (QC), phasing, and imputation of genetic data in the UK Biobank have been described elsewhere [27].

In this analysis, we excluded participants if they (1) did not have individual genotype array data, (2) withdrew from the cohort, (3) did not pass genetic QC, or (4) did not have vegetable intake data. In genetic QC, we excluded participants if (1) the self-reported sex was different from the genetic sex, (2) the sex chromosome karyotypes were putatively different from XX or XY, (3) there were outliers in heterozygosity and missing rates, indicating the sample genotypes were of poor quality, (4) they were of non-European genetic ethnicity, and (5) genetic relatedness was found with other participants in the UK Biobank (Supplementary Figure S2).

The health status of participants was followed-up via linkage to national death registries (NHS Digital for participants in England and Wales; and NHS Central Registry for participants in Scotland) and hospitalization databases (the National Health Service [NHS] Hospital Episode Statistics for participants in England; the Scottish Morbidity Record for participants in Scotland; and the Patient Episode Database for participants in Wales). At the time of this study, the death registries captured records through 28 February 2021, and the hospitalization databases captured records through 31 March 2021 for participants from England and Scotland and 28 February 2018 for participants from Wales. Diagnosis of cardiovascular outcomes was ascertained by mapping relevant codes from the International Classification of Disease (ICD) versions 9 and 10 in the death registry and hospitalization records. We used the following ICD 10 codes: *I21-I25* for CHD; *I60-I61* and *I63-I64* for stroke; *I63-I64* for ischemic stroke; *I50*, *I11.0*, *I13.0*, and *I13.2* for heart failure (HF); and *I48* for atrial fibrillation (AF). The equivalent ICD-9 codes used are shown in Supplementary Table S3. 

#### 2.2.2. Statistical Analysis

Unweighted polygenic risk scores (PRSs) for cooked and raw vegetable intake were calculated by summing the number of vegetable intake-increasing alleles carried by a participant and dividing it by the total number of SNPs. We estimated the associations between PRSs and population baseline characteristics by fitting linear regressions of PRSs on the baseline characteristics (age, sex, body mass index, physical activity, alcohol drinking, smoking, systolic blood pressure, diastolic blood pressure, red meat intake, processed meat intake, and oily fish intake), and using the *p*-value for the overall model fit as the *p*-value for the potential association. 

MR estimates were obtained using the two-stage least square method, in which two regressions were fitted. In the first stage, we fitted a multivariate linear regression model of the two PRSs on cooked and raw vegetable intake for participants without cardiovascular diseases (non-cases), adjusted for sex, age, age square, assessment center, genotype batch, and the first 20 genetic principal components [28,29]. From this first-stage regression, we obtained the genetically predicted cooked and raw vegetable intake. In the second stage, we fitted a logistic regression model of the genetically predicted cooked and raw vegetable intake on the outcomes, adjusted for the same covariates as in the first-stage regression. 

For sensitivity analysis, we fitted a Cox model in the second stage after excluding participants with CVDs at recruitment. More details are shown in the Supplementary methods. As secondary analysis, we performed univariable one-sample MR analysis for cooked and raw vegetable intake separately, using a similar two-stage least square method. In the first stage, we fitted a linear model for vegetable intake and obtained the genetically predicted values; in the second stage, a logistic regression of the predicted vegetable intake was fitted on the outcomes. The same covariates were adjusted as in the multivariable analysis. 

### 2.3. Two-Sample MR

#### 2.3.1. Data Source

For two-sample MR, we used summary-level GWAS statistics from the *CARDIoGRAMplusC4D* consortium for CHD (with 60,801 cases) [30], the *MEGASTROKE* consortium for stroke and ischemic stroke (with 40,585 and 34,217 cases, respectively) [31], the *HERMES* consortium for heart failure (with 47,309 cases) [32], and the *Nielson 2018* study for atrial fibrillation (with 60,662 cases) [33]. For replication, we used the summary-level GWAS data of the five cardiovascular outcomes from the FinnGen consortium (release 5) [34], using the following FinnGen endpoint codes: “*I9_CHD*” for CHD, “*I9_STR_SAH*” for stroke, “*I9_STR_EXH*” for ischemic stroke, “*I9_HEARTFAIL_NS*” for HF, and “*I9_AF*” for AF, respectively. All of these GWAS were performed in unrelated individuals of predominantly European ancestry. The *CARDIoGRAMplusC4D*, *MEGASTROKE*, and FinnGen consortia had no sample overlap with the UK Biobank, while the *HERMES* consortium and *Nielson 2018* study had 40% and 38% sample overlap with the UK Biobank, respectively. The basic characteristics of these GWAS are shown in Supplementary Table S1.

#### 2.3.2. Statistical Analysis

In multivariable two-sample MR, we included 43 (15 + 28) SNPs but further removed three duplicate SNPs. The remaining 40 SNPs were not in linkage disequilibrium. The associations of each SNP with cooked and raw vegetable intake were extracted from the GWAS conducted by Canela-Xandri et al. [22] (Supplementary Table S4), while the associations with the outcomes were extracted from the relevant outcome GWAS data. For the SNPs that could not be matched to outcomes in the GWAS, we first tried to identify proper proxy SNPs in linkage disequilibrium (r^2^ > 0.80, distance < 500 kb); if no proper proxy was identified, the unmatched SNPs were removed from further analysis. Finally, 39 SNPs were used in the analysis of the FinnGen-derived GWAS data (*rs11608727* was not matched); otherwise, all 40 SNPs were used. 

Summary-level association statistics for each SNP were orientated across different GWAS so that their effect estimates were aligned on the same alleles [35]. The inverse variance-weighted method was performed to estimate the associations between vegetable intake and the outcomes [36]. 

As secondary analysis, we performed univariable two-sample MR, in which 15 and 28 SNPs were used for cooked and raw vegetable intake, respectively. We used the inverse variance-weighted method, while sensitivity analyses were performed using alternative approaches, including the weighted median and MR-Egger methods. The weighted median method can generate reliable effect estimates when at least 50% of SNPs are valid instruments [37]. The MR-Egger method can detect and correct for possible directional pleiotropy [37]. Pleiotropy was examined using the MR-Egger intercept test, with a *p*-value < 0.05 suggesting the presence of directional pleiotropy, in which case the MR-PRESSO method [38] was used to examine the effect of pleiotropy. The MR-PRESSO method can detect outlier SNPs and provide effect estimates after removing outliers. 

### 2.4. Meta-Analysis

We combined the two-sample and one-sample MR estimates via meta-analysis for separate univariable and multivariable MR. A random effects model was used for the primary analysis, while a fixed effects model was used for the sensitivity analysis. The I^2^ statistic was calculated to quantify heterogeneity, with I^2^ > 50% indicating the presence of high heterogeneity. Since the HERMES consortium and *Nielson 2018* study had sample overlap with the UK Biobank, and one-sample MR estimation tends to overestimate associations [39], we performed a sensitivity meta-analysis by excluding the one-sample MR estimates.

The effects were quantified using the odds ratio (OR) and its 95% confidence interval (CI), reflecting risk change in the outcome for a lifelong increase in vegetable intake of one daily serving. Bonferroni correction was applied to control multiple comparisons for two exposures and five outcomes, α = 0.05/(2 × 5) = 0.005. The statistical tests were two-sided, with a *p*-value < 0.005 considered as a conservative level of statistical significance, and a *p*-value between 0.005 and 0.05 considered as suggestive evidence.

### 2.5. Cardiometabolic Risk Factors for Exploratory Mechanisms

We performed two-sample MR on cardiometabolic risk factors to explore potential mechanisms. SNPs that were biologically associated with the metabolism of lipids, glucose, or protein were further removed, leaving 9 and 19 SNPs for cooked and raw vegetable intake, which explained 0.5% and 2.3% of phenotypic variance, respectively. The outcomes of interest included total cholesterol (TC, mg/dL), total triglyceride (TG, mg/dL), low-density lipoprotein cholesterol (LDL, mg/dL), high-density lipoprotein cholesterol (HDL, mg/dL), body mass index (BMI, kg/m^2^), systolic blood pressure (SBP, mmHg), diastolic blood pressure (DBP, mmHg), pulse pressure (PP, the difference between SBP and DBP, mmHg), fasting insulin (FI, pmol/L), fasting glucose (FG, mmol/L), glycated hemoglobin (HbA1c, %) and 2-hour glucose after oral glucose tolerance test (OGTT, mmol/L).

We used the summary-level GWAS statistics from the Global Lipids Genetics Consortium (GLGC) for lipids-related outcomes (TC, TG, LDL, HDL) [40], *Locke 2015* for BMI [41], the Meta-Analyses of Glucose and Insulin-related traits Consortium (MAGIC) for glycemic traits (FG, FI, OGTT, HbA1c) [42], and the International Consortium of Blood Pressure (ICBP) for blood pressure measures (SBP, DBP, PP) [43], respectively. These GWAS had no sample overlap with the UK Biobank. All of these GWAS were conducted in unrelated individuals of European ancestry, and adjusted for sex, age, age square, genetic principle components, and other study-specific covariates. More details on these GWAS are summarized in Supplementary Table S1. Briefly, GLGC [40] included 188,578 individuals who were not on lipid-lowering treatment, and blood lipid levels were measured after >8 h of fasting. The *Locke 2015* GWAS [41] included 339,224 individuals. MAGIC [42] included 200,622, 151,013, 63,396, and 146,806 individuals for analyses of FG, FI, OGTT, and HbA1c, respectively. FI was natural log-transformed. Participants in MAGIC were excluded if they had a diagnosis of diabetes, were on anti-diabetic medication, or had abnormal glycemic or insulin levels (FG > 7 mmol/L, OGTT > 11.1 mmol/L, HbA1c > 6.5%). ICBP [43] included 150,134 individuals. BMI was additionally adjusted for in the GWAS of FG, FI, OGTT, SBP, DBP, and PP. We performed univariable and multivariable MR using the inverse variance-weighted method for primary analysis, while the median-based and MR-Egger methods were used for sensitivity analyses.

All analyses were performed in R (version 4.1.1) using the “*MendelianRandomization*” package (version 0.5.1), “*MR-PRESSO*” package (version 1.0), and “*meta*” package (version 5.0-1). 

## 3. Results

The average F-statistic values were 29 (range 18 to 48) for the SNPs associated with cooked vegetable intake and 30 (range 18 to 46) for the SNPs associated with raw vegetable intake, respectively, suggesting good instrument strength (Table 1).

### 3.1. One-Sample MR

In one-sample MR, 361,797 UK Biobank participants were included, with 37,014 cases of CHD, 9298 cases of stroke, 7264 cases of ischemic stroke, 11,773 cases of HF, and 25,915 cases of AF recorded during 12.1 years of follow up. The mean age was 56.9 (standard deviation (SD) 7.9) years and 55.0% were women. The mean values of cooked and raw vegetable intake were 2.74 (1.77) and 2.19 (1.98) heaped tablespoons per day, respectively (Supplementary Table S5). The correlation between cooked and raw vegetable intake was low (Pearson correlation coefficient = 0.30). The mean PRSs for cooked and raw vegetable intake were 1.10 (0.16) and 1.06 (0.12), respectively (Supplementary Figure S3). PRSs were strongly associated with actual vegetable intake (*p* < 2 × 10^−16^) and not associated with age, sex, body mass index, physical activity, smoking, drinking, blood pressure, red meat intake, or processed meat intake (Supplementary Table S6). The F-statistic values for the cooked and raw vegetable intake PRSs were 67 (range 62 to 70) and 314 (range 289 to 322), respectively (Supplementary Table S7).

**Figure 2:**
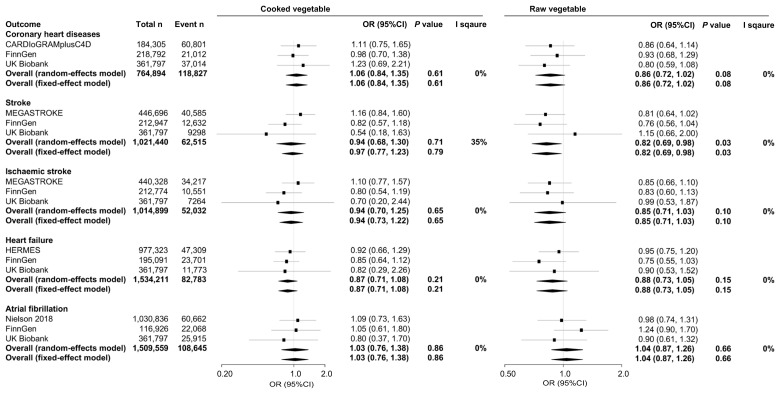
Associations between genetically predicted vegetable intake and cardiovascular risk in meta-analysis of two- and one-sample multivariable Mendelian randomization. OR (95%CI): odds ratio (95% confidence interval). MR estimates in the UK Biobank were obtained from one-sample analysis, otherwise two-sample analysis. UKB: UK Biobank. CARDIoGRAMplusC4D: Coronary ARtery DIsease Genome-wide Replication and Meta-analysis plus The Coronary Artery Disease Genetics) consortium. MEGASTROKE: MEGASTROKE consortium. HERMES: HEart failure Molecular Epidemiology for therapeutic targetS consortium.

In multivariable one-sample analysis, we did not find significant evidence for associations between genetically predicted vegetable intake and cardiovascular outcomes (Figure 2). Univariable analyses and subsequent sensitivity analyses also generated nonsignificant evidence for associations (Supplementary Tables S7 and S8).

### 3.2. Two Sample MR

In multivariable two-sample MR analysis mutually adjusted for cooked and raw vegetable intake, we observed null evidence for associations between raw vegetable intake and CHD, stroke, ischemic stroke, and HF, consistent across different data sources (Figure 2). The univariable analysis showed similarly null evidence for most of the associations (Supplementary Table S9).

The potential presence of directional pleiotropy was found in cooked vegetable intake and ischemic stroke in *FinnGen* (*p*-value for MR-Egger intercept = 0.05) and the association between raw vegetable intake and AF in *FinnGen* (*p*-value for MR-Egger intercept = 0.01). However, MR-PRESSO analysis detected zero and one outlier SNP (*rs62380935*), respectively, and removing the outlier yielded very similar results. The weighted median method generated consistent results with the inverse variance-weighted estimates (Supplementary Table S9).

### 3.3. Meta-Analysis

Meta-analysis of the two-sample and one-sample multivariable MR estimates revealed suggestive evidence for an inverse association between genetically predicted raw vegetable intake and stroke (OR (95%CI): 0.82 (0.69, 0.98), *p* = 0.03), but it failed to pass the Bonferroni-corrected significance level (0.005). The associations of raw vegetable intake with other CVD outcomes were directionally inverse (except AF): CHD (0.86 (0.72, 1.02), *p* = 0.08), ischemic stroke (0.85 (0.71, 1.03), *p* = 0.10), and HF (0.88 (0.73, 1.05), *p* = 0.15) (Figure 2). Sensitivity analysis by excluding the one-sample estimate from the UK Biobank consistently showed suggestive evidence for an association between raw vegetable intake and stroke risk (0.79 (0.66, 0.95), *p* = 0.01) (Supplementary Table S10). Meta-analysis of univariable estimates showed nonsignificant evidence for associations between raw vegetable intake with the outcomes (Supplementary Table S11, Supplementary Figure S4). We did not find significant evidence for associations between cooked vegetable intake and CVDs. There was no evidence of heterogeneity in the meta-analysis; the fixed and random effects models produced similar results. 

### 3.4. Cardiometabolic Risk Factors for Exploratory Mechanisms

**Figure 3:**
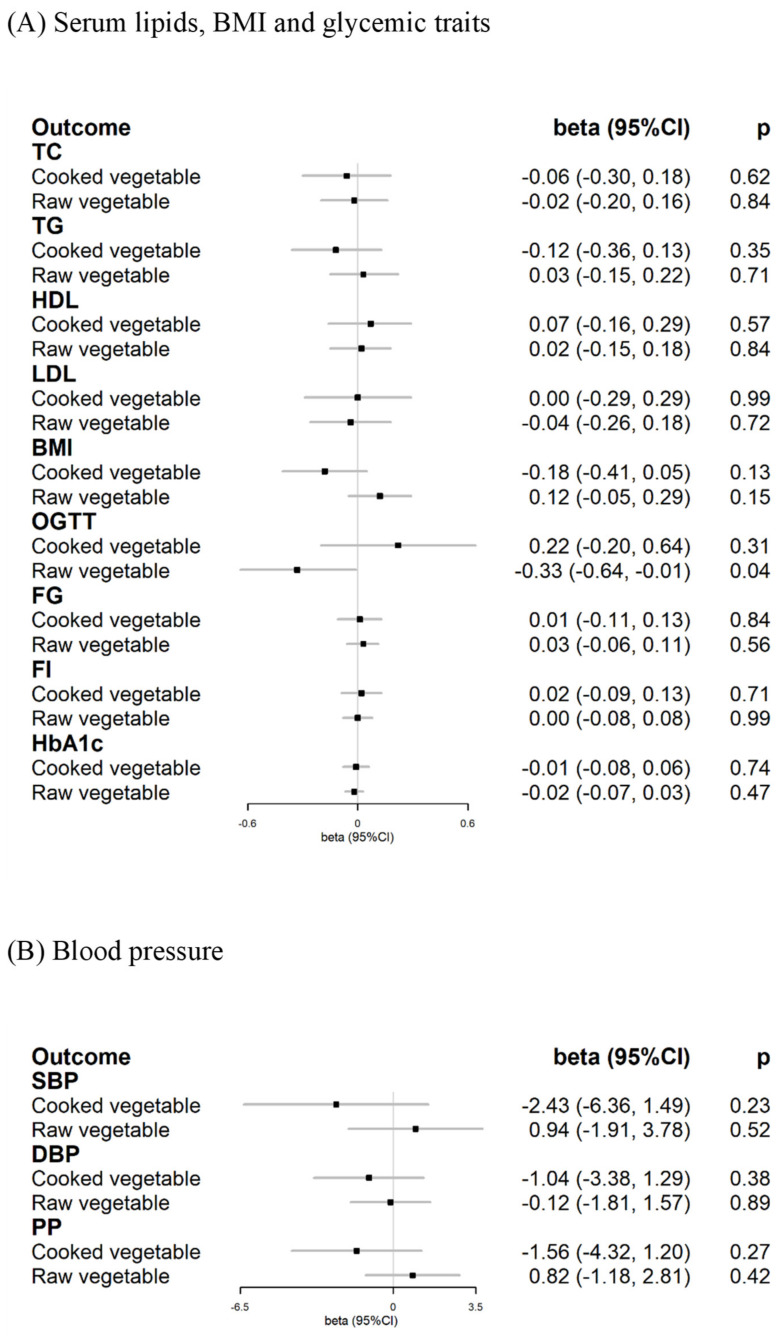
Associations between genetically predicted vegetable intake and serum lipids, BMI, glycemic traits, and blood pressure in multivariable Mendelian randomization. (**A**) Serum lipids, BMI and glycemic traits; (**B**) Blood pressure. TC: total cholesterol, mg/dL. TG: triglyceride, mg/dL. HDL: high-density lipoprotein, mg/dL. LDL: low-density lipoprotein, mg/dL. BMI: body mass index, kg/m^2^. OGTT: 2-hour glucose after oral glucose tolerance test, mmol/L. FG: fasting glucose, mmol/L. FI: fasting insulin, mmol/L. HbA1c: glycated hemoglobin, %. SBP: systolic blood pressure, mmHg. DBP: diastolic blood pressure, mmHg. PP: pulse pressure, mmHg.

Nine and 19 SNPs were included in this analysis, with average F-statistic values of 30 and 29 for SNPs of cooked and raw vegetable intake, respectively, suggesting good instrument strength (Supplementary Table S12). Univariable and multivariable MR showed similar results (Supplementary Tables S13 and S14, Supplementary Figure S5). Overall, in the primary multivariable MR using the inverse variance-weighted method, genetically determined vegetable intake was not associated with serum lipids, BMI, glycemic traits, or BP (Figure 3, Supplementary Table S14). For each one serving increase in cooked vegetable intake, the *beta*-values (95%CI) for TC, BMI, FG, and SBP were −0.06 (−0.30, 0.18; *p* = 0.62), −0.18 (−0.41, 0.05; *p* = 0.13), 0.01 (−0.11, 0.13; *p* = 0.84), and −2.43 (−6.36, 1.49; *p* = 0.23), respectively. For each serving increase in raw vegetable intake, the *beta*-values (95%CI) for TC, BMI, FG, and SBP were −0.02(−0.20, 0.16; *p* = 0.84), 0.12 (−0.05, 0.29; *p* = 0.15), 0.03 (−0.06, 0.11; *p* = 0.56), and 0.94 (−1.91, 3.78; *p* = 0.52), respectively. A suggestive association between raw vegetable intake and reduced OGTT was observed (−0.33 [−0.64, −0.01; *p* = 0.04]), but it did not pass Bonferroni correction. The MR-Egger and median-based methods showed similar results of null associations, and the MR-Egger intercept test suggested no evidence of horizontal pleiotropy (Supplementary Table S14).

## 4. Discussion

This MR analysis of 1.2 million participants generally demonstrated overall null evidence for associations between genetically predicted vegetable intake with CHD, HF, and AF. Mechanism analyses provided further null evidence for associations with cardiometabolic risk factors, including serum lipids, BMI, blood pressure, and glycemic measures. 

Previous meta-analyses of cohort studies have found that higher vegetable intake was associated with reduced CVD risks [10,11]. However, causal inference has been difficult because residual confounding is ubiquitous in observational research, while randomized controlled trials with large sample size and long follow-up times for capturing clinical outcomes have been sparse and inconclusive [44,45]. The findings in this MR study seem contradictory to observational evidence, and residual confounding is likely to be one of the reasons [16,46]. A previous analysis of 400,000 UK Biobank participants [16] estimated that residual confounding accounted for about 80–90% of the observational associations between vegetable intake and CVD outcomes, and this percentage was likely to be higher providing further adjustment for unobserved confounders and/or more accurate measurement of the confounders. Although MR has been regarded as a promising approach in causal inference, its validity depends on some underlying assumptions. First, that the instrumental variables are associated with at least one of the exposure variables. Second, that there are no unmeasured confounders of the associations between genetic variants and outcomes. Third, that the genetic variants affect the outcomes only through their effect on the exposure of interest [47].

For the first assumption, we selected the SNPs that were associated with cooked or raw vegetable intake at a genome-wide association level in three GWAS. The PRSs in one-sample MR were highly associated with the observed phenotypes (*p* < 2 × 10^−16^). High F-statistic values also indicated their high strength as instrument variables. Additionally, a number of the selected SNPs were biologically associated with vegetable intake via their regulatory effect on olfactory receptors, gastrointestinal health, tooth health, and metabolism of lipids/protein/glucose. 

For the second assumption, we searched the Phenoscanner v2 database for any phenotypes associated with the SNPs, and excluded the SNPs that were associated with potential confounders, including alcohol drinking, smoking, blood pressure, and adiposity. In one-sample MR, the PRSs were not associated with common cardiovascular risk factors, such as alcohol drinking, smoking, physical activity, blood pressure, obesity, red meat intake, and processed meat intake. Although the PRSs were associated with oily fish intake, adjustment for it in the two-stage least square analysis did not change the results (Supplementary Table S7). For the third assumption, the MR-Egger intercept test did not show strong evidence of directional pleiotropy for most of the analyses; MR-PRESSO analysis generated similar results to the primary inverse variance-weighted estimates. Sensitivity analyses using the median-based and MR-Egger methods also generated consistent results. Therefore, the three assumptions were plausibly satisfied in our study. 

Nevertheless, completely excluding the possibility of true associations between vegetable intake and CVDs is unlikely based on the null evidence in this study. Selecting appropriate genetic instruments for exposure is difficult [19,48]. Firstly, the genetic architecture of consuming vegetables, as a behavior, is not fully understood. Although we have discovered some selected SNPs associated with traits that may determine vegetable intake, the biological functions of other SNPs remain unclear. Secondly, the phenotypic variance explained by the SNPs was small (0.8% for cooked vegetable intake and 2.4% for raw vegetable intake), which must be acknowledged, even though it is common for behavioral traits.

Dietary-derived antioxidants, especially vitamin C, vitamin E, retinol, carotene, and lycopene, have been proposed as the major mechanisms for the observational protective effects [49] and are valid biomarkers reflecting vegetable consumption level [50]. Prior MR studies on these antioxidants showed similar null associations. Kobylecki et al. used one SNP, *rs33972313*, in the *SLC23A1* gene region, which encodes sodium-dependent vitamin C transporter 1, as the genetic instrument for serum vitamin C, and reported that vitamin C was not associated with incident CHD nor all-cause mortality in a cohort of 100,000 Danish participants [51]. Zhu et al., using 9 SNPs associated with serum circulating vitamin C, further found null genetic associations with a range of cardiovascular risk factors and diseases, including CHD, stroke, HF, AF, blood pressure, obesity, and serum lipids [52]. Luo et al. investigated five antioxidants, i.e., vitamin C, vitamin E, retinol, carotene, and lycopene, in both absolute circulating levels and relative metabolite levels, and found null evidence for any associations with incident CHD [53]. Similarly, Martens et al. found that the five antioxidants were not associated with stroke [54]. However, these studies focused on a single nutrient and ignored the potential additive and synergistic effects of various antioxidants, as well as those between antioxidants and minerals, fiber, and other phytochemicals, which could be a more plausible mechanism for CVD risk reduction due to increased vegetable intake [49,55]. Therefore, future research is required for causal inference and mechanism investigation. 

Extra caution should be taken when interpreting the findings. The MR estimates reflect the lifetime risk change in the outcomes due to solely increasing vegetable intake by one daily serving, while all other risk factors remain unchanged [56]. It is assumed that all other risk factors for CVDs are fixed, including socioeconomic, lifestyle, and other dietary factors. However, diet is always complex, characterized by the intake of many different kinds of food and substitutions between them, where high consumption of vegetables is associated with lower intake of other food given the relative stability of an individual’s calorie intake [57,58]. The general population should be cautious about replacing different kinds of food items, but this is beyond the scope of our study and warrants future research on dietary patterns that describe the overall diet. 

We observed a potential inverse association between raw vegetable intake and incident stroke, which passed the conventional significance level (0.05) but failed to pass the Bonferroni-corrected significance level (0.01). This is in line with a previous study of 20,000 individuals [59]. If this association is a true effect, this may indicate potentially different health effects of cooked and raw vegetables on stroke, which has been suggested in previous observational studies [12,14]. Nevertheless, this remains unclear and requires future research.

This study had some limitations. First, the biological mechanisms behind the SNPs and vegetable-eating behavior are not completely understood. Second, although we differentiated cooked and raw vegetable intake in this study, these phenotypes are still a mix of different vegetable kinds and cooking methods (for cooked vegetables). It may be valuable to further differentiate vegetable kinds and cooking methods in future studies. Third, the dietary intake was measured in the UK biobank, a cohort based on England, Wales, and Scotland, so the findings may be more generalizable to populations that consume similar types of vegetables (e.g., carrots, broccoli, spinach, peppers) and use similar cooking methods. Fourth, vegetable intake was measured using a self-reporting questionnaire in the UK Biobank. It was not directly validated against biomarkers, although comparison to 24-h recall assessment showed good agreement. Fifth, our analysis was confined to a population of European ancestry, which reduced population stratification bias but may limit its generalizability to populations of other ethnicities. Sixth, our one-sample estimates may be vulnerable to “winner’s curse” as the UK Biobank was used for both selecting the SNPs and estimating the associations of interest, which may bias the one-sample estimates towards the null; however, the two-sample MR estimates generated consistent results, so the potential effect of “winner’s curse” on our overall estimate should be minor.

## 5. Conclusions

We performed MR analyses and their meta-analysis, and found null evidence for associations between genetically predicted cooked and raw vegetable intake with CHD, AF, HF, and a range of cardiometabolic risk factors, but we observed potential evidence for an inverse association between raw vegetable intake and risk of stroke. The possibility of true associations between vegetable intake and CVDs cannot be completely ruled out because of the difficulty in identifying statistically and biologically plausible genetic proxies for dietary factors. More investigation is warranted for causal inference in nutritional research.

## Figures and Tables

**Table 1:** Characteristics of the SNPs associated with cooked and raw vegetable intake.

SNPs	Chromo-Some	Position	Effect Allele	Other Allele	Effect Allele Frequency	Beta	Standard Error	*p*-Value	F-Statistic	Nearest Gene
Cooked vegetable intake										
rs1534749	1	190028576	C	T	0.470	−0.017	0.003	2.15 × 10^−7^	26.895	*BRINP3*
rs3001363	1	154125067	T	C	0.489	−0.018	0.003	2.68 × 10^−8^	30.929	*NUP210L*
rs113993820	2	102766634	T	G	0.019	−0.061	0.012	3.53 × 10^−7^	25.939	*IL1R1*
rs2102738	2	172525884	C	A	0.172	−0.023	0.004	1.15 × 10^−7^	28.098	*DYNC1I2*
rs442291	2	79676305	C	T	0.389	0.023	0.003	4.03 × 10^−12^	48.113	*CTNNA2*
rs17653477	3	71170319	G	A	0.031	−0.046	0.009	1.31 × 10^−6^	23.415	*FOXP1*
rs10020708	4	178097496	A	C	0.494	−0.015	0.003	2.52 × 10^−6^	22.156	*NEIL3*
rs17714824	5	158254070	T	G	0.175	0.024	0.004	1.36 × 10^−8^	32.245	*EBF1, FABP6*
rs33947258	5	141194870	A	C	0.261	0.023	0.004	5.01 × 10^−10^	38.673	*PCDH1*
rs12190945	6	84162042	G	A	0.296	−0.015	0.004	2.46 × 10^−5^	17.791	*ME1*
rs6975898	7	4540687	G	T	0.376	−0.017	0.003	5.61 × 10^−7^	25.044	*FOXK1*
rs11995369	8	89649177	C	T	0.202	0.023	0.004	2.40 × 10^−8^	31.142	*MMP16*
rs10156602	9	96345328	G	A	0.362	0.020	0.003	4.66 × 10^−9^	34.329	*PHF2*
rs10161952	13	59474383	C	A	0.313	−0.017	0.004	2.60 × 10^−6^	22.093	*DIAPH3*
rs6420335	13	69556727	G	C	0.467	−0.018	0.003	3.07 × 10^−8^	30.665	*KLHL1*
Raw vegetable intake										
rs11209780	1	71876652	A	G	0.216	−0.025	0.005	1.33 × 10^−7^	27.821	*NEGR1*
rs3001363	1	154125067	T	C	0.489	−0.025	0.004	9.01 × 10^−11^	42.028	*NUP210L*
rs3828120	1	82434387	A	T	0.328	0.023	0.004	1.20 × 10^−8^	32.494	*ADGRL2*
rs11125813	2	59991047	A	G	0.219	0.023	0.005	7.26 × 10^−7^	24.546	*BCL11A*
rs4281874	2	176451226	T	C	0.265	0.023	0.004	1.14 × 10^−7^	28.127	*LNPK*
rs442291	2	79676305	C	T	0.389	0.023	0.004	2.49 × 10^−9^	35.549	*CTNNA2*
rs78940216	2	27153318	A	G	0.111	−0.030	0.006	9.70 × 10^−7^	23.988	*DPYSL5*
rs12630752	3	44303185	G	A	0.234	−0.023	0.005	3.73 × 10^−7^	25.829	*TOPAZ1*
rs17075255	5	164759108	T	C	0.234	−0.028	0.005	6.98 × 10^−10^	38.027	*MAT2B*
rs2915858	5	166542621	G	A	0.432	0.022	0.004	8.80 × 10^−9^	33.092	*TENM2*
rs62380935	5	137723585	G	A	0.215	0.026	0.005	3.44 × 10^−8^	30.442	*KDM3B*
rs9359954	6	92318594	G	T	0.479	0.017	0.004	5.93 × 10^−6^	20.509	*MAP3K7*
rs57221424	7	35215670	G	C	0.322	0.024	0.004	3.28 × 10^−9^	35.011	*DPY19L2*
rs6958768	7	77773693	C	A	0.167	−0.026	0.005	6.78 × 10^−7^	24.677	*MAGI2*
rs13255011	8	35051793	T	C	0.479	0.021	0.004	6.54 × 10^−8^	29.197	*UNC5D*
rs13267577	8	4847469	T	C	0.381	−0.024	0.004	1.05 × 10^−9^	37.239	*CSMD1*
rs1520919	8	64696606	A	G	0.299	−0.026	0.004	7.55 × 10^−10^	37.873	*YTHDF3*
rs687135	9	37257202	T	C	0.454	−0.021	0.004	3.55 × 10^−8^	30.386	*ZCCHC7*
rs7857380	9	128555022	C	A	0.365	−0.027	0.004	1.26 × 10^−11^	45.877	*PBX3*
rs67497633	10	103815495	A	G	0.169	0.029	0.005	1.69 × 10^−8^	31.827	*ARMH3*
rs11608727	12	110060984	G	T	0.196	−0.026	0.005	6.77 × 10^−8^	29.131	*MVK*
rs10161952	13	59474383	C	A	0.313	−0.021	0.004	1.95 × 10^−7^	27.082	*DIAPH3*
rs77797947	13	56160164	A	C	0.033	0.051	0.012	9.28 × 10^−6^	19.655	*PRR20A*
rs9323534	14	20586432	T	C	0.433	−0.022	0.004	2.00 × 10^−8^	31.493	*OR4K17*
rs1437761	15	97010698	C	T	0.249	−0.023	0.004	1.13 × 10^−7^	28.132	*NR2F2*
rs956362	15	35927655	G	A	0.212	0.020	0.005	2.40 × 10^−5^	17.841	*DPH6*
rs2447090	17	2298974	G	A	0.361	−0.018	0.004	4.81 × 10^−6^	20.911	*MNT*
rs6079589	20	14850762	T	C	0.218	−0.025	0.005	3.09 × 10^−8^	30.652	*MACROD2*

The associations between the SNPs and vegetable intake were obtained from the GWAS conducted by Canela-Xandri et al. (2018) [22]. SNP: single nucleotide polymorphism. The 15 eligible SNPs associated with cooked vegetable intake explained 0.8% of phenotypic variance, while the 28 SNPs associated with raw vegetable intake explained 2.4% of phenotypic variance. Information on nearest gene, relevant functions, and enrichment are obtained from the Ensembl database (http://www.ensembl.org/Homo_sapiens/Info/Index, accessed on 15 July 2023), FIVEx database (https://fivex.sph.umich.edu/, accessed on 15 July 2023), GTExPortal database (https://gtexportal.org/home/, accessed on 15 July 2023), and GeneCards database (https://www.genecards.org/, accessed on 15 July 2023).

## Data Availability

The summary-level GWAS data can be downloaded at http://www.cardiogramplusc4d.org/steering-committee/ (accessed on 15 July 2023) for the CARDIoGRAMplusC4D consortium; at https://www.megastroke.org/ (accessed on 15 July 2023) for the MEGASTROKE consortium; at https://cvd.hugeamp.org/downloads.html#summary (accessed on) for the HERMES consortium; at https://magicinvestigators.org/ (accessed on 15 July 2023) for MAGIC; at http://lipidgenetics.org/ (accessed on 15 July 2023) for GLGC; at https://portals.broadinstitute.org/collaboration/giant/index.php/GIANT_consortium (accessed on 15 July 2023) for Locke 2015; and at http://geneatlas.roslin.ed.ac.uk/ (accessed on 15 July 2023) for the GWAS conducted by Canela-Xandri et al. (2018) [22]. FinnGen summary-level GWAS data are available at https://www.finngen.fi/fi (accessed on 15 July 2023) upon application. UK Biobank individual-level data are available at https://www.ukbiobank.ac.uk/ (accessed on 15 July 2023) upon application. For ICBP GWAS data, please send a request to ICBP. Analytic R codes for this study are available upon request.

## Supplementary Materials

The following supporting information can be downloaded at: https://www.mdpi.com/article/10.3390/nu15173682/s1, Supplementary methods; Figure S1: The process of SNP selection; Figure S2: Flowchart of participant selection in UK Biobank for one-sample Mendelian randomization analysis; Figure S3: Histograms for the distribution of vegetable intake genetic risk scores (GRS); Figure S4: Associations between vegetable intake and cardiovascular risk in meta-analysis of univariable Mendelian randomization; Figure S5: Forest plots for the leave-one-out analysis for selected genetic associations between vegetable intake and cardiometabolic risk factors; Table S1: Characteristics of the included genome-wide association studies; Table S2: Biological functions of the SNPs associated with cooked and raw vegetable intake; Table S3: The ICD9 and ICD10 codes to confirm the cardiovascular outcomes in UK Biobank; Table S4: Characteristics of the included SNPs included in two-sample multivariable Mendelian randomization; Table S5: Baseline characteristics of the UK Biobank participants included in one-sample Mendelian randomization; Table S6: Associations (showing p values) between vegetable intake genetic risk scores and baseline characteristics in UK Biobank; Table S7: Associations between vegetable intake and cardiovascular risk in multivariable one-sample Mendelian randomization in UK Biobank; Table S8: Associations between vegetable intake and cardiovascular risk in univariable one-sample Mendelian randomization in UK Biobank; Table S9: Associations between vegetable intake and cardiovascular risk in univariable two-sample Mendelian randomization in UK Biobank; Table S10: Associations between vegetable intake and cardiovascular risk in meta-analysis excluding UK Biobank effect estimates; Table S11: Associations between vegetable intake and cardiovascular risk in meta-analysis of univariable Mendelian randomization; Table S12: The basic characteristics of the SNPs for cooked and raw vegetable intake included in Mendelian randomization analysis for cardiometabolic risk factors; Table S13: Association between vegetable intake and cardiometabolic risk factors in univariable Mendelian randomization; Table S14: Association between vegetable intake and body mass index, serum lipids in multivariable Mendelian randomization.
