# Supplementary material for: Genetically Predicted Vegetable Intake and Cardiovascular Diseases and Risk Factors: An Investigation with Mendelian Randomization
Source: Nutrients. 2023 Aug 22;15(17):3682. doi: 10.3390/nu15173682 (PMC10490460; doi:10.3390/nu15173682)
Supplement: Supplementary file 1 [file nutrients-15-03682-s001.zip › nutrients-2571060-supplementary.pdf]

Supplementary methods
Supplementary table S1: Characteristics of the included genome-wide association studies.
Supplementary figure S1: The process of SNP selection.
Supplementary table S2: Biological functions of the SNPs associated with cooked and raw vegetable intake.
Supplementary figure S2: Flowchart of participant selection in UK Biobank for one-sample Mendelian randomization analysis.
Supplementary table S3: The ICD9 and ICD10 codes to confirm the cardiovascular outcomes in UK Biobank.
Supplementary table S4: Characteristics of the included SNPs included in two-sample multivariable Mendelian randomization.
Supplementary table S5: Baseline characteristics of the UK Biobank participants included in one-sample Mendelian randomization.
Supplementary figure S3: Histograms for the distribution of vegetable intake genetic risk scores (GRS).
Supplementary table S6: Associations (showing p values) between vegetable intake genetic risk scores and baseline characteristics in UK Biobank.
Supplementary table S7: Associations between vegetable intake and cardiovascular risk in multivariable one-sample Mendelian randomization in UK Biobank.
Supplementary table S8: Associations between vegetable intake and cardiovascular risk in univariable one-sample Mendelian randomization in UK Biobank.
Supplementary table S9: Associations between vegetable intake and cardiovascular risk in univariable two-sample Mendelian randomization in UK Biobank.
Supplementary table S10: Associations between vegetable intake and cardiovascular risk in meta-analysis excluding UK Biobank effect estimates.
Supplementary table S11: Associations between vegetable intake and cardiovascular risk in meta-analysis of univariable Mendelian randomization.
Supplementary figure S4: Associations between vegetable intake and cardiovascular risk in meta-analysis of univariable Mendelian randomization.
Supplementary table S12: The basic characteristics of the SNPs for cooked and raw vegetable intake included in Mendelian randomization analysis for cardiometabolic risk factors.
Supplementary table S13: Association between vegetable intake and cardiometabolic risk factors in univariable Mendelian randomization.
Supplementary figure S5: Forest plots for the leave-one-out analysis for selected genetic associations between vegetable intake and cardiometabolic risk factors.
Supplementary table S14: Association between vegetable intake and body mass index, serum lipids in multivariable Mendelian randomization.

Supplementary methods

Covariate measurement in UK Biobank

UK Biobank measured a range of baseline characteristics. Physical activity was assessed using the International Physical Activity Questionnaire and categorized into low, moderate, and high activity levels [1]. Briefly, high level of physical activity is defined as vigorous-intensity activity on at least 3 days achieving a minimum total physical activity of at least 1500 metabolic equivalent of task (MET)-minutes/week, or 7 days of any combination of walking, moderate-intensity or vigorous-intensity activities achieving a minimum total physical activity of at least 3000 MET-minutes/week; moderate level is defined as 3 or more days of vigorous-intensity activity of at least 20 minutes per day, or 5 or more days of moderate-intensity activity and/or walking of at least 30 minutes per day, or 5 or more days of any combination of walking, moderate-intensity or vigorous intensity activities achieving a minimum total physical activity of at least 600 MET-minutes/week; otherwise low level. Smoking and drinking status were coded as never, previous or current users.

Blood pressure was measured on their left forearm with Omron 705 IT Electronic Blood Pressure Monitor when participants were at resting status. Two readings with more than one minute lap were taken and averaged as the final reading. A sphygmomanometer with an inflatable cuff is used in conjunction with a stethoscope only when the electronic monitor failed to produce a reading. Body mass index (BMI) was calculated as body weight (kilograms) divided by height squared (meters). Drinking and smoking status was self-reported and categorized into previous users, current users and never users.

Frequency of intake of oily fish (< 1, 1, ≥ 2 times/week), processed meat (0, ≤ 1, ≥ 2 times per week) and red meat were measured with baseline dietary questionnaire. Intake of red meat was the sum of pork, beef and mutton/lamb, and was transformed into a continuous variable using the method proposed by Bradbury et al. [2]. All answers of "unknown", "do not know", "prefer not to say" for each categorical variable were combined into one category of "unknown" and included in analyses as a separate category.

Multivariable one-sample Mendelian randomization (MR)

The effects of cooked and raw vegetable intake were assessed jointly, so their effects were adjusted for each other. Two-stage least square method was used. In the first stage, we fitted a multivariate regression in controls only (participants without the outcome):

$$(cooked\ vegetable\ intake_{observed}, raw\ vegetable\ intake_{observed}) \sim$$
$$\beta_0 + \beta_1 * GRS_{cooked} + \beta_2 * GRS_{raw} + \beta_3 * covariates \quad \text{(S1)}$$

in which the covariates include sex, age, age square, genotype batch, assessment centre and first 20 genetic principle components. From this fitted model, we obtained the genetically predicted cooked and raw vegetable intakes, and used them in the second stage of logistic regression, as below:

$$CVD\ outcome \sim$$
$$\theta_0 + \theta_1 * cooked\ vegetable\ intake_{predicted} + \theta_2 * raw\ vegetable\ intake_{predicted} + \theta_3 * covariates \quad \text{(S2)}$$

in which the covariates include sex, age, age square, genotype batch, assessment centre and first 20 genetic principle components. The coefficients θ_1 and θ_2 are the MR estimates for cooked vegetable intake and raw vegetable intake, respectively.

Univariable one-sample MR

The effects of raw and cooked vegetable intakes were assessed in separate models, so their effects were not adjusted for each other. Two-stage least square method was used to assess the association, in which two stages of regression were performed. Take cooked vegetable intake as an example, the first stage regression was fitted as a linear regression:

$$cooked\ vegetable\ intake_{observed} \sim$$
$$\beta_0 + \beta_1 * GRS_{cooked} + \beta_2 * covariates \text{ (S3)}$$

in which the covariates include sex, age, age square, genotype batch, assessment centre and first 20 genetic principle components. From this fitted model (1), we obtained the genetically predicted cooked vegetable intake, and use it in the second stage, which was a logistic regression, as below:

$$CVD\ outcome \sim$$
$$\theta_0 + \theta_1 * cooked\ vegetable\ intake_{predicted} + \theta_2 * covariates \text{ (S4)}$$

in which the covariates include sex, age, age square, genotype batch, assessment centre and first 20 genetic principle components. The coefficient of the predicted cooked vegetable intake, θ_1, was the final MR estimate for the effect. Using similar method we estimated the effect of raw vegetable intake.

Sensitivity analysis for one-sample MR

In the above models, the adjustment of sex, age, age square, genotype batch, assessment centre and genetic principle components was recommended [3]. As we observed that in UK Biobank the GRS of vegetable intake were associated with physical activity level, smoking, drinking and oily-fish intake, we additionally adjusted for these covariates in the second stage regression, as one of the sensitivity analyses.

We also fitted Cox regression in the second stage regression, instead of logistic regression, in sensitivity analysis. To fit the cox model, we excluded the participants with baseline cardiovascular diseases. Participants were censored at date of hospitalization, date of death, or the last of follow-up (31 March 2021 for participants from England and Scotland, and 28 February 2018 for participants from Wales). Similar one-sample and two-sample analysis were performed.

Some formulas

Proportion of the phenotypic variance explained by a SNP:

$$R^2 = 2 * EAF * (1 - EAF) * beta^2$$

in which, EAF is the effect allele frequency, and beta is the association between the SNP and the phenotype in standard deviation unit.

F statistics

$$F = (\frac{beta}{se})^2$$

In which, beta is the association between the SNP and the phenotype, and se is the according standard error.

References:

1 World Health Organization. Global Physical Activity Questionnaire Analysis Guide. https://www.who.int/ncds/surveillance/steps/resources/GPAQ_Analysis_Guide.pdf

2 Bradbury KE, Young HJ, Guo W, *et al.* Dietary assessment in UK Biobank: an evaluation of the performance of the touchscreen dietary questionnaire. *J Nutr Sci* 2018;**7**:e6. doi:10.1017/jns.2017.66

3 Burgess S, Thompson SG. Multivariable Mendelian Randomization: The Use of Pleiotropic Genetic Variants to Estimate Causal Effects. *American Journal of Epidemiology* 2015;**181**:251–60. doi:10.1093/aje/kwu283

Supplementary table S1: Characteristics of included genome-wide association studies.

Phenotype	Sample size	Population	Phenotype definition	GWAS adjustment
**Exposure: vegetable intake**				
Niarchou 2020	335576	European-ancestry individuals in UK Biobank	Cooked and raw vegetable intakes as continuous variables, truncated at 3.5 standard deviations.	Age, sex, month of assessment, assessment centre, batch, 100 genetic PCs
Cole 2020	455146	European-ancestry individuals in UK Biobank	Cooked and raw vegetable intakes as continuous variables, using inverse rank normal transformation.	Age, sex
Canela-Xandri 2018	452264	European-ancestry individuals in UK Biobank	Cooked and raw vegetable intakes as continuous variables, truncated at 10 standard deviations.	Age, sex, array batch, assessment centre, age square, and the leading 20 genetic PCs
**Outcome: Coronary heart disease**				
CARDIoGRAMplusC4D consortium	184305 (60801 /123504)*	European-ancestry individuals	Coronary artery diseases	--
FinnGen	218792 (21012 /197780)	European-ancestry individuals	Major coronary heart disease event. (I9_CHD)<br>ICD10: I2[1-5]|I46|R96|R98.<br>ICD9: 41[0-4]|798.<br>ICD8: 41[0-4]|798.	Age, sex, ancestry principal components and genotyping batch
**Stroke**				

MEGASTROKE consortium	446696 (40585 /406111) *	European-ancestry individuals	Any stroke	Age, sex, ancestry principle components 1-10, study site
FinnGen	212947 (12632 /200315) *	European-ancestry individuals	Stroke, including subarachnoid haemorrhage. (I9_STR_SAH) ICD10: I60|I61|I63|I64. ICD9:430|431|4330A|4331A|4339A|4340A|4341A|4349A|436. ICD8: 430|431|433|434|436.	Age, sex, ancestry principal components and genotyping batch
**Ischaemic stroke**				
MEGASTROKE consortium	440328 (34217 /406111) *	European-ancestry individuals	Any ischemic stroke	Age, sex, ancestry principle components 1-10, study site
FinnGen	212774 (10551 /202223) *	European-ancestry individuals	Ischaemic stroke, excluding all haemorrhages. (I9_STR_EXH) ICD10: I63|I64. ICD9: 4330A|4331A|4339A|4340A|4341A|4349A|436. ICD8: 433|434|436.	Age, sex, ancestry principal components and genotyping batch
**Heart failure**				
HERMES consortium §	977323 (47309 /930014) *	European-ancestry individuals	Heart failure (I9_HEARTFAIL_NS)	Age, sex, ancestry principal components
FinnGen	195091 (23701 /171390) *	European-ancestry individuals	Heart failure. ICD10: I50|I110|I130|I132. ICD9: 4029B|428	Age, sex, ancestry principal components and genotyping batch

**Atrial Fibrillation**				
Nielson 2018 #	1030836 (60662 /970174) *	European-ancestry individuals	Atrial fibrillation (I9_AF)	--
FinnGen	116926 (22068 /94858) *	European-ancestry individuals	Atrial fibrillation. ICD10: I48. ICD9: 4273.	Age, sex, ancestry principal components and genotyping batch
**Cardiometabolic risk factors**				
**TC, mg/dL**				
GLGC 2013 (PMID 24097068)	188578	European-ancestry individuals (not on lipid-lowering medication)	Blood lipid levels were measured after > 8 hours of fasting.	Age, sex, age square, genetic principle components
**HDL, mg/dL**				
GLGC 2013 (PMID 24097068)	188578	European-ancestry individuals (not on lipid-lowering medication)	Blood lipid levels were measured after > 8 hours of fasting	Age, sex, age square, genetic principle components
**LDL, mg/dL**				
GLGC 2013 (PMID 24097068)	188578	European-ancestry individuals (not on lipid-lowering medication)	Blood lipid levels were measured after > 8 hours of fasting	Age, sex, age square, genetic principle components
**TG, mg/dL**				

GLGC 2013 (PMID 24097068)	188578	European-ancestry individuals (not on lipid-lowering medication)	Blood lipid levels were measured after > 8 hours of fasting	Age, sex, age square, genetic principle components
**BMI, kg/m²**				
Locke 2015 (PMID 25673413)	339224	European-ancestry individuals	Measured or self-reported weight in kilogram divided by height square in metre	Age, sex, age square, genetic principle components, study specific covariates
**FG, mmol/L**				
MAGIC 2021 (PMID 34059833)	200622	European-ancestry individuals without diabetes	mmol/L, obtained from whole blood, corrected to plasma levels using a correction factor 1.13	Age, sex, age square, BMI, genetic principle components, study-specific covariates
**FI, pmol/L**				
MAGIC 2021 (PMID 34059833)	151013	European-ancestry individuals without diabetes	pmol/L, obtained from whole blood, corrected to plasma levels using a correction factor 1.13. Natural log-transformed in analysis.	Age, sex, age square, BMI, genetic principle components, study-specific covariates
**OGTT, mmol/L**				
MAGIC 2021 (PMID 34059833)	63396	European-ancestry individuals without diabetes	mmol/L, obtained 2 hours after a glucose challenge using an oral glucose-tolerance test	Age, sex, age square, BMI, genetic principle components, study-specific covariates
**HbA1c, %**				
MAGIC 2021 (PMID 34059833)	146806	European-ancestry individuals without diabetes	As percentage	Age, sex, age square, genetic principle components, study-specific covariates
**SBP, mmHg**				

ICBP 2017 (PMID 28739976)	150134	European-ancestry individuals	15 mmHg added to the raw SBP value for antihypertensive medication users	Age, sex, age square, BMI, genetic principle components, study-specific covariates
**DBP, mmHg**				
ICBP 2017 (PMID 28739976)	150134	European-ancestry individuals	10 mmHg added to the raw SBP value for antihypertensive medication users	Age, sex, age square, BMI, genetic principle components, study-specific covariates
**PP, mmHg**				
ICBP 2017 (PMID 28739976)	150134	European-ancestry individuals	Difference between SBP and DBP	Age, sex, age square, BMI, genetic principle components, study-specific covariates

*: case number/control number.

§: the HERMES consortium has 40% sample overlap with UK Biobank.

#: the Nielson 2018 study has 38% sample overlap with UK Biobank.

TC: total cholesterol. TG: triglyceride. HDL: high density lipoprotein. LDL: low density lipoprotein. BMI: body mass index. OGTT: 2-hour glucose after oral glucose tolerance test. FG: fasting glucose. FI: fasting insulin. HbA1c: glycated haemoglobin. SBP: systolic blood pressure. DBP: diastolic blood pressure. PP: pulse pressure. GLGC: Global Lipids Genetics Consortium. MAGIC: the Meta-Analyses of Glucose and Insulin-related traits Consortium. ICBP: International Consortium for Blood Pressure.

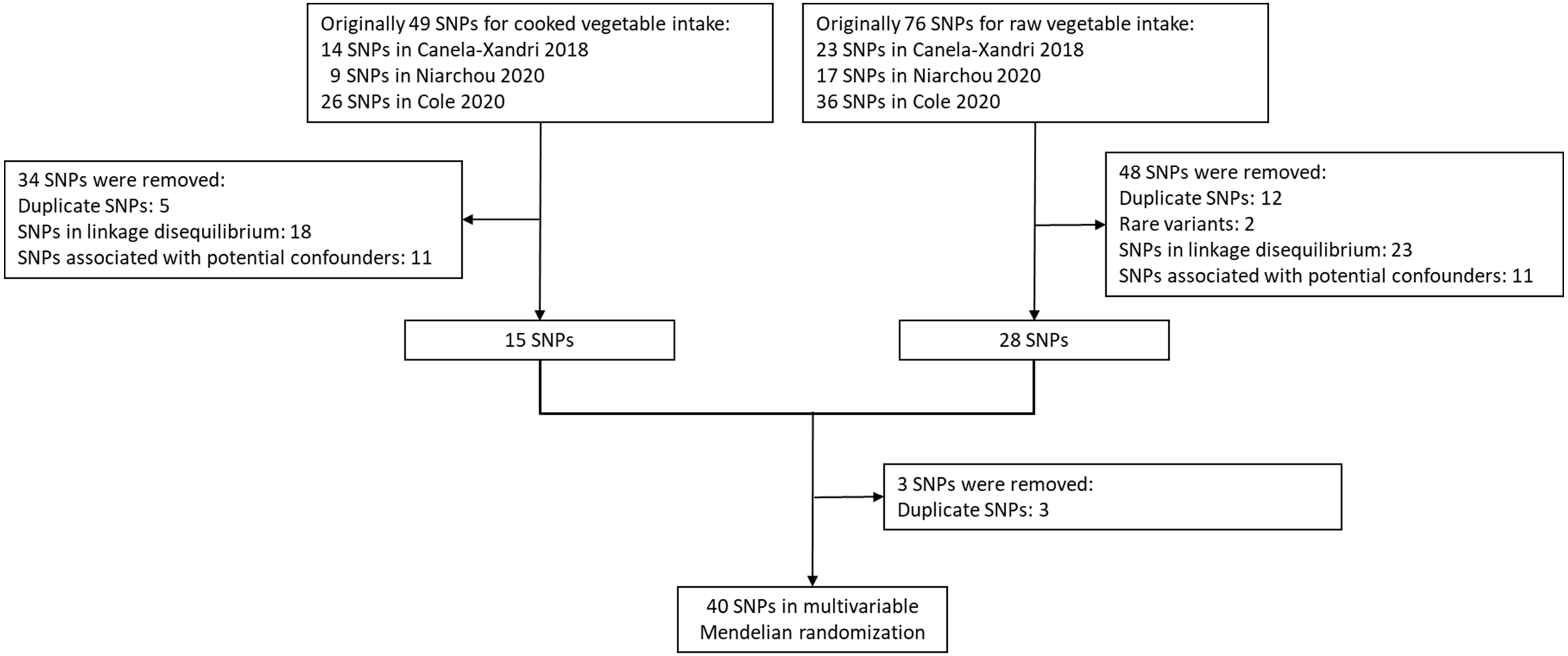

Supplementary figure S1: The process of SNP selection for cooked and raw vegetable intake.
Criteria for linkage disequilibrium: r^2 0.001 and distance 10 000 kb.
Potential confounders: blood pressure, smoking, drinking, and adiposity (body weight, body mass index, body fat mass, body fat percentage, waist circumference, hip circumference, waist-to-hip ratio). The associated phenotypes for each SNP was searched in Phenoscanner v2 database (http://www.phenoscanner.medschl.cam.ac.uk/) at genome-wide significance level ($p < 5 * 10^{-8}$). SNP: single nucleotide polymorphism.

Supplementary table S2: Biological functions of the SNPs associated with cooked and raw vegetable intake.

SNPs	Chromo-some	Position	Effect allele	Other allele	Effect allele frequency	Nearest gene	Relevant functions	Enrichment in gastrointestinal tract organs and glands
**Cooked vegetable intake**								
rs1534749	1	190028576	C	T	0.470	BRINP3	Unclear	No
rs3001363	1	154125067	T	C	0.489	NUP210L	Unclear	Yes
rs113993820	2	102766634	T	G	0.019	IL1R1	Unclear	Yes
rs2102738	2	172525884	C	A	0.172	DYNC1I2	Unclear	Yes
rs442291	2	79676305	C	T	0.389	CTNNA2	Unclear	No
rs17653477	3	71170319	G	A	0.031	FOXP1	Unclear	Yes
rs10020708	4	178097496	A	C	0.494	NEIL3	Unclear	Yes
rs17714824	5	158254070	T	G	0.175	EBF1, FABP6	lipid metabolism	Yes
rs33947258	5	141194870	A	C	0.261	PCDH1	lipid metabolism	Yes
rs12190945	6	84162042	G	A	0.296	ME1	lipid metabolism	Yes
rs6975898	7	4540687	G	T	0.376	FOXK1	protein metabolism, glucose metabolism	Yes
rs11995369	8	89649177	C	T	0.202	MMP16	Unclear	Yes
rs10156602	9	96345328	G	A	0.362	PHF2	bone/tooth health	Yes
rs10161952	13	59474383	C	A	0.313	DIAPH3	Unclear	No
rs6420335	13	69556727	G	C	0.467	KLHL1	Unclear	No
**Raw vegetable intake**								
rs11209780	1	71876652	A	G	0.216	NEGR1	protein metabolism	Yes
rs3001363	1	154125067	T	C	0.489	NUP210L	Unclear	Yes
rs3828120	1	82434387	A	T	0.328	ADGRL2	Unclear	No
rs11125813	2	59991047	A	G	0.219	BCL11A	Unclear	No
rs4281874	2	176451226	T	C	0.265	LNPK	Unclear	No
rs442291	2	79676305	C	T	0.389	CTNNA2	Unclear	No
rs78940216	2	27153318	A	G	0.111	DPYSL5	lipid metabolism	Yes
rs12630752	3	44303185	G	A	0.234	TOPAZ1	Unclear	Yes
rs17075255	5	164759108	T	C	0.234	MAT2B	lipid metabolism	No

rs2915858	5	166542621	G	A	0.432	TENM2			Yes
rs62380935	5	137723585	G	A	0.215	KDM3B	Unclear		Yes
rs9359954	6	92318594	G	T	0.479	MAP3K7	Unclear		No
rs57221424	7	35215670	G	C	0.322	DPY19L2	glucose metabolism, protein metabolism		Yes
rs6958768	7	77773693	C	A	0.167	MAGI2	Unclear		Yes
rs13255011	8	35051793	T	C	0.479	UNC5D	Unclear		Yes
rs13267577	8	4847469	T	C	0.381	CSMD1	Unclear		No
rs1520919	8	64696606	A	G	0.299	YTHDF3	Unclear		Yes
rs687135	9	37257202	T	C	0.454	ZCCHC7	Unclear		Yes
rs7857380	9	128555022	C	A	0.365	PBX3	Unclear		Yes
rs67497633	10	103815495	A	G	0.169	ARMH3	Crohn's disease		Yes
rs11608727	12	110060984	G	T	0.196	MVK	gastrointestinal dismay, lipid metabolism		No
rs10161952	13	59474383	C	A	0.313	DIAPH3	Unclear		No
rs77797947	13	56160164	A	C	0.033	PRR20A	Unclear		No
rs9323534	14	20586432	T	C	0.433	OR4K17	olfactory receptor		No
rs1437761	15	97010698	C	T	0.249	NR2F2	Unclear		Yes
rs956362	15	35927655	G	A	0.212	DPH6	Unclear		Yes
rs2447090	17	2298974	G	A	0.361	MNT	Unclear		Yes
rs6079589	20	14850762	T	C	0.218	MACROD2	protein metabolism		No

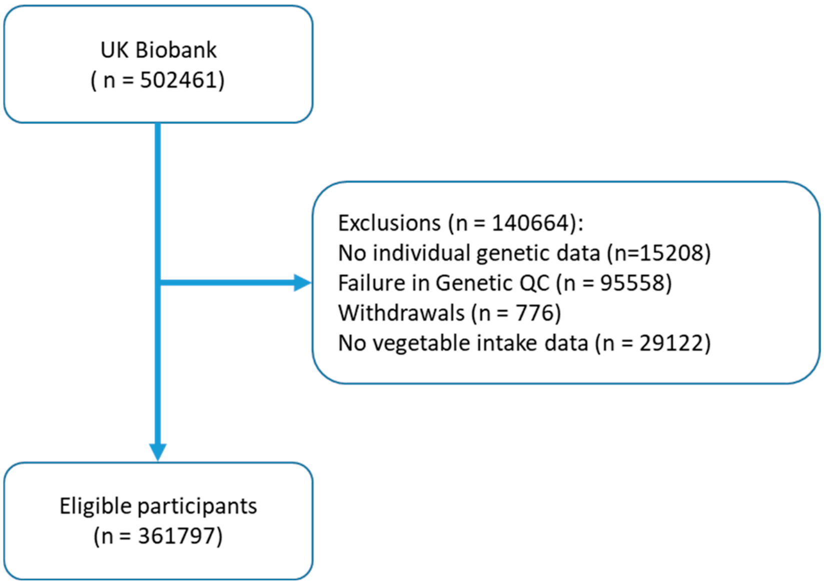

Supplementary figure S2: Flowchart of participant selection in UK Biobank for one-sample Mendelian randomization analysis.
Genetic QC: genetic quality control, in which we excluded participants if (1) the reported sex was different from their genetic sex, (2) the sex chromosome karyotypes are putatively different from XX or XY, (3) outliers in heterozygosity and missing rates, indicating the sample genotypes are of poor quality, (4) non-Caucasian genetic ethnicity, (5) genetic relatedness with other participants in UK Biobank.

Supplementary table S3: The ICD9 and ICD10 codes to confirm the cardiovascular outcomes in UK Biobank.

Cardiovascular outcomes	ICD 9 codes	ICD 10 codes
Coronary heart disease	410-414, 798	I21-I25
Stroke	430, 431, 434, 436	I60, I61, I63, I64
Ischaemic stroke	434, 436	I63, I64
Heart failure	4029, 428	I50, I11.0, I13.0, I13.2
Atrial fibrillation	4273	I48

Supplementary table S4: Characteristics of the included SNPs included in two-sample multivariable Mendelian randomization.

SNP	Chr	Position	EA	OA	EAF	Gene	Phenotype*	Cooked vegetable intake				Raw vegetable intake			
								beta	se	p	F	beta	se	p	F
rs10020708	4	178097496	C	A	0.506	--	cooked	0.015	0.003	2.52E-06	22.156	0.004	0.004	2.93E-01	1.104
rs10156602	9	96345328	G	A	0.362	PHF2	cooked	0.020	0.003	4.66E-09	34.329	0.011	0.004	7.70E-03	7.103
rs10161952	13	59474383	A	C	0.687	--	raw	0.017	0.004	2.60E-06	22.093	0.021	0.004	1.95E-07	27.082
rs11125813	2	59991047	A	G	0.219	--	raw	0.008	0.004	3.55E-02	4.423	0.023	0.005	7.26E-07	24.546
rs11209780	1	71876652	G	A	0.784	NEGR1	raw	0.002	0.004	6.58E-01	0.196	0.025	0.005	1.33E-07	27.821
rs113993820	2	102766634	G	T	0.981	IL1R1	cooked	0.061	0.012	3.53E-07	25.939	0.041	0.014	3.30E-03	8.634
rs11608727	12	110060984	T	G	0.804	--	raw	0.015	0.004	2.29E-04	13.581	0.026	0.005	6.77E-08	29.131
rs11995369	8	89649177	C	T	0.202	LOC105375630	cooked	0.023	0.004	2.40E-08	31.142	-0.003	0.005	4.72E-01	0.518
rs12190945	6	84162042	A	G	0.704	--	cooked	0.015	0.004	2.46E-05	17.791	-0.002	0.004	6.09E-01	0.262
rs12630752	3	44303185	A	G	0.766	TOPAZ1	raw	0.012	0.004	1.84E-03	9.706	0.023	0.005	3.73E-07	25.829
rs13255011	8	35051793	T	C	0.479	--	raw	0.013	0.003	7.64E-05	15.646	0.021	0.004	6.54E-08	29.197
rs13267577	8	4847469	C	T	0.619	CSMD1	raw	0.007	0.003	3.38E-02	4.506	0.024	0.004	1.05E-09	37.239
rs1437761	15	97010698	T	C	0.751	--	raw	0.011	0.004	4.20E-03	8.193	0.023	0.004	1.13E-07	28.132
rs1520919	8	64696606	G	A	0.701	LINC01289	raw	0.011	0.004	2.72E-03	8.986	0.026	0.004	7.55E-10	37.873

rs1534749	1	190028576	T	C	0.530	--	cooked	0.017	0.003	2.15E-07	26.895	0.010	0.004	6.54E-03	7.396
rs17075255	5	164759108	C	T	0.766	--	raw	0.001	0.004	7.72E-01	0.084	0.028	0.005	6.98E-10	38.027
rs17653477	3	71170319	A	G	0.969	FOXP1	cooked	0.046	0.009	1.31E-06	23.415	-0.004	0.011	7.16E-01	0.132
rs17714824	5	158254070	T	G	0.175	EBF1	cooked	0.024	0.004	1.36E-08	32.245	0.018	0.005	4.64E-04	12.254
rs2102738	2	172525884	A	C	0.828	--	cooked	0.023	0.004	1.15E-07	28.098	0.015	0.005	2.84E-03	8.911
rs2447090	17	2298974	A	G	0.639	MNT	raw	0.008	0.003	1.96E-02	5.451	0.018	0.004	4.81E-06	20.911
rs2915858	5	166542621	G	A	0.432	TENM2	raw	0.015	0.003	7.48E-06	20.067	0.022	0.004	8.80E-09	33.092
rs3001363	1	154125067	C	T	0.511	NUP210L	cooked, raw	0.018	0.003	2.68E-08	30.929	0.025	0.004	9.01E-11	42.028
rs33947258	5	141194870	A	C	0.261	LOC107986454	cooked	0.023	0.004	5.01E-10	38.673	0.007	0.004	1.22E-01	2.397
rs3828120	1	82434387	A	T	0.328	ADGRL2	raw	0.009	0.003	6.58E-03	7.384	0.023	0.004	1.20E-08	32.494
rs4281874	2	176451226	T	C	0.265	--	raw	0.002	0.004	5.69E-01	0.325	0.023	0.004	1.14E-07	28.127
rs442291	2	79676305	C	T	0.389	--	cooked, raw	0.023	0.003	4.03E-12	48.113	0.023	0.004	2.49E-09	35.549
rs57221424	7	35215670	G	C	0.322	DPY19L2P1	raw	0.008	0.004	1.73E-02	5.668	0.024	0.004	3.28E-09	35.011
rs6079589	20	14850762	C	T	0.782	MACROD2	raw	0.005	0.004	1.66E-01	1.919	0.025	0.005	3.09E-08	30.652
rs62380935	5	137723585	G	A	0.215	KDM3B	raw	0.009	0.004	2.08E-02	5.341	0.026	0.005	3.44E-08	30.442
rs6420335	13	69556727	C	G	0.533	--	cooked	0.018	0.003	3.07E-08	30.665	0.018	0.004	4.01E-06	21.262

rs67497633	10	103815495	A	G	0.169	ARMH3	raw	0.005	0.004	2.70E-01	1.217	0.029	0.005	1.69E-08	31.827
rs687135	9	37257202	C	T	0.546	ZCCHC7	raw	0.013	0.003	9.26E-05	15.282	0.021	0.004	3.55E-08	30.386
rs6958768	7	77773693	A	C	0.833	MAGI2	raw	0.019	0.004	1.70E-05	18.501	0.026	0.005	6.78E-07	24.677
rs6975898	7	4540687	T	G	0.624	--	cooked	0.017	0.003	5.61E-07	25.044	0.006	0.004	1.59E-01	1.984
rs77797947	13	56160164	A	C	0.033	--	raw	0.002	0.010	8.12E-01	0.057	0.051	0.012	9.28E-06	19.655
rs7857380	9	128555022	A	C	0.635	PBX3	cooked, raw	0.013	0.003	7.07E-05	15.791	0.027	0.004	1.26E-11	45.877
rs78940216	2	27153318	G	A	0.889	DPYSL5	raw	0.017	0.005	1.41E-03	10.195	0.030	0.006	9.70E-07	23.988
rs9323534	14	20586432	C	T	0.567	OR4K17	raw	0.008	0.003	2.16E-02	5.277	0.022	0.004	2.00E-08	31.493
rs9359954	6	92318594	G	T	0.479	--	raw	0.007	0.003	2.37E-02	5.118	0.017	0.004	5.93E-06	20.509
rs956362	15	35927655	G	A	0.212	DPH6-DT	raw	0.012	0.004	2.30E-03	9.294	0.020	0.005	2.40E-05	17.841

The associations between the SNPs and vegetable intake were obtained from the Canela-Xandri 2018 study. SNP: single nucleotide polymorphism. Chr: chromosome. EA: effect allele. OA: other allele. EAF: effect allele frequency.

Supplementary table S5: Baseline characteristics of the UK Biobank participants included in one-sample Mendelian randomization.

	Men (n = 162841)	Women (n = 198956)	Total (n = 361797)
Age, years	57.21 (8.07)	56.73 (7.90)	56.94 (7.98)
Cooked vegetable intake, tablespoons/day	2.76 (1.90)	2.72 (1.65)	2.74 (1.77)
Cooked vegetable intake GRS	1.10 (0.16)	1.10 (0.16)	1.10 (0.16)
Raw vegetable intake, tablespoons/day	1.97 (1.94)	2.36 (1.98)	2.19 (1.98)
Raw vegetable intake GRS	1.06 (0.12)	1.06 (0.12)	1.06 (0.12)
Body mass index, kg/m^2	27.84 (4.21)	27.01 (5.10)	27.38 (4.74)
Systolic blood pressure, mmHg	143.28 (18.49)	137.66 (20.23)	140.20 (19.66)
Diastolic blood pressure, mmHg	84.12 (10.53)	80.67 (10.50)	82.23 (10.65)
Red meat intake, times/week	2.27 (1.46)	1.99 (1.33)	2.12 (1.40)
Processed meat intake, times/week			
< 1	7600 (4.67)	22843 (11.49)	30443 (8.42)
1	83468 (51.3)	134333 (67.58)	217801 (60.25)
≥ 2	71645 (44.03)	41595 (20.93)	113240 (31.33)
Oily fish intake, times/week			
< 1	72503 (44.7)	82387 (41.53)	154890 (42.96)
1	60753 (37.46)	79211 (39.93)	139964 (38.82)
≥ 2	28943 (17.84)	36771 (18.54)	65714 (18.23)
Physical activity level			
Low	25504 (18.22)	28244 (18.04)	53748 (18.13)
Moderate	53778 (38.42)	67284 (42.98)	121062 (40.83)
High	60705 (43.36)	61022 (38.98)	121727 (41.05)
Drinking status			
Never drinker	2649 (1.63)	8497 (4.27)	11146 (3.08)
Previous drinker	5125 (3.15)	7043 (3.54)	12168 (3.37)
Current drinker	154965 (95.22)	183273 (92.18)	338238 (93.55)
Smoking status			
Never smoker	79199 (48.8)	118132 (59.57)	197331 (54.72)
Previous smoker	64571 (39.79)	63850 (32.2)	128421 (35.61)
Current smoker	18525 (11.41)	16336 (8.24)	34861 (9.67)

GRS: genetic risk score. Physical activity level was measured by the International Physical Activity Questionnaire.

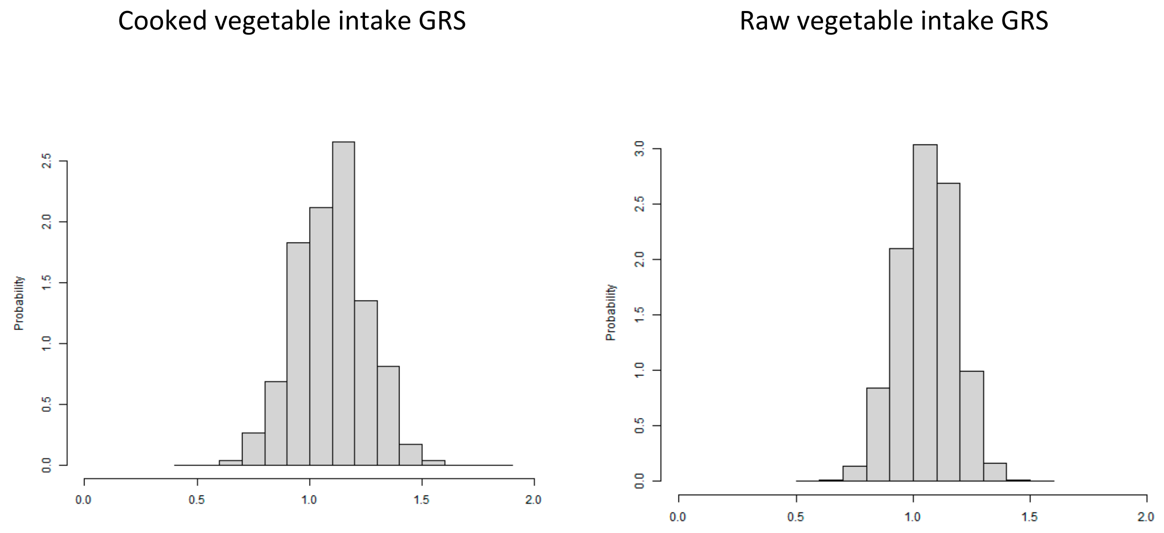

Supplementary figure S3: Histograms for the distribution of vegetable intake genetic risk scores (GRS).

Supplementary table S6: Associations (showing p values) between vegetable intake genetic risk scores and baseline characteristics in UK Biobank

	Cooked vegetable intake GRS	Raw vegetable intake GRS
Age	0.152	0.660
Sex	0.394	0.008
Body mass index	0.188	0.103
Physical activity level	0.014	0.044
Drinking status	0.271	0.004
Smoking status	0.099	0.002
Systolic blood pressure	0.578	0.147
Diastolic blood pressure	0.081	0.111
Red meat intake	0.152	0.470
Processed meat intake	0.861	0.202
Oily fish intake	0.0004 #	$1.44 * 10^{-11}$ #
Fruit intake	$3.40 * 10^{-5}$ #	0.0002 #
Cooked vegetable intake	$< 2.00 * 10^{-16}$ #	$1.86 * 10^{-15}$ #
Raw vegetable intake	$2.96 * 10^{-6}$ #	$< 2.00 * 10^{-16}$ #

The table shows the p value for the overall fitting of the linear models of each baseline characteristic on vegetable intake GRS (genetic risk score).
#: association significant under Bonferroni correction (to control multiple comparison: p = 0.05/28 = 0.0017).

Supplementary table S7: Associations between vegetable intake and cardiovascular risk in multivariable one-sample Mendelian randomization in UK Biobank.

			Cooked vegetable		Raw vegetable	
	Total n	Event n	F statistics	OR (95% CI)	F statistics	OR (95% CI)
**Primary analysis**						
Coronary heart disease	361797	37014	68.67	1.23 (0.69, 2.21)	288.74	0.80 (0.59, 1.08)
Stroke	361797	9298	70.17	0.54 (0.18, 1.63)	319.57	1.15 (0.66, 2.00)
Ischaemic stroke	361797	7264	70.27	0.70 (0.20, 2.44)	322.39	0.99 (0.53, 1.87)
Heart failure	361797	11773	68.03	0.82 (0.29, 2.26)	321.19	0.90 (0.53, 1.52)
Atrial fibrillation	361797	25915	62.42	0.80 (0.37, 1.70)	318.80	0.90 (0.61, 1.32)
**Sensitivity analysis 1**						
Coronary heart disease	340615	21819	68.67	1.44 (0.73, 2.82)	288.74	0.80 (0.56, 1.14)
Stroke	345189	6425	70.17	0.67 (0.19, 2.38)	319.57	0.97 (0.50, 1.90)
Ischaemic stroke	345562	5082	70.27	1.05 (0.25, 4.46)	322.39	0.80 (0.38, 1.71)
Heart failure	344735	8243	68.03	0.90 (0.28, 2.85)	321.19	0.80 (0.44, 1.46)
Atrial fibrillation	342070	18624	62.42	0.77 (0.35, 1.71)	318.80	0.98 (0.65, 1.50)
**Sensitivity analysis 2**						
Coronary heart disease	361797	37014	68.67	1.25 (0.69, 2.23)	288.74	0.80 (0.59, 1.08)
Stroke	361797	9298	70.17	0.56 (0.18, 1.67)	319.57	1.15 (0.66, 2.01)
Ischaemic stroke	361797	7264	70.27	0.71 (0.20, 2.51)	322.39	1.00 (0.53, 1.88)
Heart failure	361797	11773	68.03	0.82 (0.30, 2.28)	321.19	0.91 (0.54, 1.53)
Atrial fibrillation	361797	25915	62.42	0.79 (0.37, 1.70)	318.80	0.89 (0.61, 1.31)
**Sensitivity analysis 3**						
Coronary heart disease	340615	21819	68.67	1.65 (0.86, 3.16)	288.74	0.76 (0.54, 1.07)
Stroke	345189	6425	70.17	0.73 (0.22, 2.46)	319.57	1.07 (0.58, 1.98)

Ischaemic stroke	345562	5082	70.27	1.00 (0.25, 3.92)	322.39	0.91 (0.46, 1.82)
Heart failure	344735	8243	68.03	0.86 (0.30, 2.50)	321.19	0.84 (0.49, 1.46)
Atrial fibrillation	342070	18624	62.42	0.76 (0.35, 1.68)	318.80	0.98 (0.66, 1.46)

Primary analysis: The one-sample MR two-stage least squares estimates were obtained using logistic regression to estimate the association between the genetic risk score and the health outcomes.
Sensitivity analysis 1: The one-sample MR two-stage least squares estimates were obtained using cox regression to estimate the association between the genetic risk score and the health outcomes, after excluding participants with baseline cardiovascular diseases.
Sensitivity analysis 2: The one-sample MR two-stage least squares estimates were obtained using logistic regression to estimate the association between the genetic risk score and the health outcomes, with additional adjustment for potential residual confounder (oily fish and fruit intake).
Sensitivity analysis 3: The one-sample MR two-stage least squares estimates were obtained using cox regression to estimate the association between the genetic risk score and the health outcomes, after excluding participants with baseline cardiovascular diseases, with additional adjustment for potential residual confounder (oily fish and fruit intake).

Supplementary table S8: Associations between vegetable intake and cardiovascular risk in univariable one-sample Mendelian randomization in UK Biobank.

			Cooked vegetable		Raw vegetable	
	Total n	Event n	F statistics	OR (95% CI)	F statistics	OR (95% CI)
**Primary analysis**						
Coronary heart disease	361797	37014	68.67	1.09 (0.68, 1.74)	288.74	0.87 (0.71, 1.06)
Stroke	361797	9298	70.17	0.59 (0.24, 1.43)	319.57	0.92 (0.64, 1.32)
Ischaemic stroke	361797	7264	70.27	0.69 (0.25, 1.91)	322.39	0.87 (0.58, 1.31)
Heart failure	361797	11773	68.03	0.77 (0.34, 1.75)	321.19	0.83 (0.60, 1.15)
Atrial fibrillation	361797	25915	62.42	0.74 (0.42, 1.33)	318.80	0.82 (0.66, 1.03)
**Sensitivity analysis 1**						
Coronary heart disease	340615	21819	68.67	1.27 (0.74, 2.17)	288.74	0.91 (0.72, 1.16)
Stroke	345189	6425	70.17	0.66 (0.24, 1.83)	319.57	0.83 (0.54, 1.28)
Ischaemic stroke	345562	5082	70.27	0.93 (0.29, 2.95)	322.39	0.82 (0.50, 1.33)
Heart failure	344735	8243	68.03	0.80 (0.32, 2.00)	321.19	0.77 (0.52, 1.12)
Atrial fibrillation	342070	18624	62.42	0.77 (0.42, 1.41)	318.80	0.89 (0.69, 1.14)
**Sensitivity analysis 2**						
Coronary heart disease	361797	37014	68.67	1.10 (0.69, 1.76)	288.74	0.87 (0.71, 1.06)
Stroke	361797	9298	70.17	0.60 (0.25, 1.47)	319.57	0.93 (0.65, 1.34)
Ischaemic stroke	361797	7264	70.27	0.71 (0.26, 1.96)	322.39	0.88 (0.58, 1.33)
Heart failure	361797	11773	68.03	0.78 (0.35, 1.77)	321.19	0.84 (0.61, 1.16)
Atrial fibrillation	361797	25915	62.42	0.74 (0.41, 1.32)	318.80	0.81 (0.65, 1.02)
**Sensitivity analysis 3**						
Coronary heart disease	340615	21819	68.67	1.41 (0.84, 2.38)	288.74	0.91 (0.73, 1.14)
Stroke	345189	6425	70.17	0.76 (0.28, 2.04)	319.57	0.96 (0.64, 1.43)
Ischaemic stroke	345562	5082	70.27	0.95 (0.31, 2.86)	322.39	0.91 (0.58, 1.43)

Heart failure	344735	8243	68.03	0.79 (0.34, 1.85)	321.19	0.80 (0.57, 1.12)
Atrial fibrillation	342070	18624	62.42	0.75 (0.41, 1.38)	318.80	0.88 (0.70, 1.11)

Primary analysis: The one-sample MR two-stage least squares estimates were obtained using logistic regression to estimate the association between the genetic risk score and the health outcomes.
Sensitivity analysis 1: The one-sample MR two-stage least squares estimates were obtained using cox regression to estimate the association between the genetic risk score and the health outcomes, after excluding participants with baseline cardiovascular diseases.
Sensitivity analysis 2: The one-sample MR two-stage least squares estimates were obtained using logistic regression to estimate the association between the genetic risk score and the health outcomes, with additional adjustment for potential residual confounder (oily fish and fruit intake).
Sensitivity analysis 3: The one-sample MR two-stage least squares estimates were obtained using cox regression to estimate the association between the genetic risk score and the health outcomes, after excluding participants with baseline cardiovascular diseases, with additional adjustment for potential residual confounder (oily fish and fruit intake).

Supplementary table S9: Associations between vegetable intake and cardiovascular risk in univariable two-sample Mendelian randomization.

			Inverse-variance weighted		Weighted median		MR-Egger		
	No. SNP	F statistics	OR (95%CI)	P	OR (95%CI)	P	OR (95%CI)	p	p for intercept
Coronary heart disease									
**Cooked vegetable**									
CARDIoGRAMplusC4D	15	71.63	1.09 (0.80, 1.48)	0.58	1.41 (0.95, 2.08)	0.08	0.81 (0.23, 2.84)	0.75	0.64
FinnGen	15	71.63	1.07 (0.69, 1.68)	0.75	0.96 (0.55, 1.66)	0.87	0.55 (0.09, 3.40)	0.52	0.46
**Raw vegetable**									
CARDIoGRAMplusC4D	28	101.43	0.88 (0.72, 1.08)	0.21	0.82 (0.64, 1.06)	0.13	0.83 (0.20, 3.40)	0.80	0.93
FinnGen	27[#]	101.62	1.06 (0.83, 1.35)	0.65	1.10 (0.78, 1.57)	0.58	0.78 (0.14, 4.36)	0.78	0.73
Stroke									
**Cooked vegetable**									
MEGASTROKE	15	71.63	0.99 (0.74, 1.34)	0.96	0.74 (0.50, 1.07)	0.11	0.63 (0.19, 2.12)	0.46	0.45
FinnGen	15	71.63	1.03 (0.62, 1.73)	0.90	0.85 (0.47, 1.54)	0.59	4.63 (0.66, 32.77)	0.12	0.12
**Raw vegetable**									
MEGASTROKE	28	101.43	0.91 (0.77, 1.07)	0.25	0.91 (0.72, 1.14)	0.40	0.61 (0.20, 1.85)	0.38	0.47
FinnGen	27[#]	101.62	0.85 (0.65, 1.12)	0.25	0.80 (0.55, 1.14)	0.22	0.88 (0.13, 6.17)	0.90	0.97
Ischaemic stroke									
**Cooked vegetable**									
MEGASTROKE	15	71.63	0.96 (0.68, 1.36)	0.82	0.75 (0.50, 1.14)	0.18	0.38 (0.10, 1.46)	0.16	0.16
FinnGen	15	71.63	0.88 (0.51, 1.53)	0.65	0.86 (0.45, 1.63)	0.64	5.98 (0.81, 44.05)	0.08	0.05*
**Raw vegetable**									
MEGASTROKE	28	101.43	0.94 (0.79, 1.13)	0.51	1.12 (0.87, 1.44)	0.37	0.67 (0.20, 2.24)	0.52	0.57
FinnGen	27[#]	101.62	0.82 (0.62, 1.08)	0.16	0.84 (0.57, 1.24)	0.38	0.90 (0.13, 6.32)	0.92	0.92
**Heart failure**									
**Cooked vegetable**									
HERMES	15	71.63	1.38 (1.06, 1.80)	0.02	1.62 (1.15, 2.28)	0.01	1.45 (0.50, 4.16)	0.49	0.92

FinnGen	15	71.63	1.05 (0.75, 1.48)	0.76	1.17 (0.75, 1.83)	0.49	3.56 (0.98, 12.89)	0.05	0.06
**Raw vegetable**									
HERMES	28	101.43	1.09 (0.94, 1.27)	0.25	1.10 (0.90, 1.35)	0.34	0.79 (0.29, 2.18)	0.65	0.53
FinnGen	27[#]	101.62	1.14 (0.93, 1.40)	0.21	1.26 (0.94, 1.68)	0.12	0.79 (0.19, 3.34)	0.75	0.61
**Atrial fibrillation**									
**Cooked vegetable**									
Nielson	15	71.63	0.98 (0.79, 1.21)	0.84	0.87 (0.67, 1.13)	0.29	0.83 (0.35, 1.97)	0.67	0.69
FinnGen	15	71.63	1.22 (0.61, 2.47)	0.57	0.98 (0.51, 1.90)	0.96	4.56 (0.28, 75.65)	0.29	0.34
**Raw vegetable**									
Nielson 2018	28	101.43	1.03 (0.83, 1.29)	0.78	1.26 (1.02, 1.55)	0.04	0.76 (0.16, 3.54)	0.72	0.69
FinnGen	27[#]	101.62	0.92 (0.61, 1.37)	0.68	0.98 (0.64, 1.50)	0.94	0.03 (0.00, 0.45)	0.01	0.01**

*: MR PRESSO method was performed because the p for MR-Egger intercept = 0.05, which identified zero outlier SNPs, and the estimated inverse-variance weighted OR (95%CI) remained 0.88 (0.51, 1.53).

**: MR PRESSO method was performed because the p for MR-Egger intercept < 0.05, which identified 1 outlier SNP (rs62380935), and the estimated inverse-variance weighted OR (95%CI) remained 1.02 (0.72, 1.46). p = 0.10 for the difference between the original IVW estimate and the MR-PRESSO corrected estimate.

#: rs11608727 was not matched in FinnGen.

UKB: UK Biobank. CARDIoGRAMplusC4D: Coronary ARtery DIsease Genome wide Replication and Meta-analysis plus The Coronary Artery Disease Genetics) consortium. MEGASTROKE: MEGASTROKE consortium. HERMES: Heart Failure Molecular Epidemiology for Therapeutic Targets consortium. OR (95%CI): odds ratio (95% confidence interval)

Supplementary table S10: Associations between vegetable intake and cardiovascular risk in meta-analysis excluding UK Biobank effect estimates.

			Cooked vegetable			Raw vegetable		
	Total n	Event n	OR (95%CI)	I2	P*	OR (95%CI)	I2	P*
**Coronary heart diseases**								
CARDIoGRAMplusC4D	184305	60801	1.11 (0.75, 1.65)			0.86 (0.64, 1.14)		
FinnGen	218792	21012	0.98 (0.70, 1.38)			0.93 (0.68, 1.29)		
Overall (random-effects model)	403097	81813	1.03 (0.80, 1.34)	0%	0.80	0.89 (0.72, 1.10)	0%	0.28
Overall (fixed-effect model)			1.03 (0.80, 1.34)		0.80	0.89 (0.72, 1.10)		0.28
**Stroke**								
MEGASTROKE	446696	40585	1.16 (0.84, 1.60)			0.81 (0.64, 1.02)		
FinnGen	212947	12632	0.82 (0.57, 1.18)			0.76 (0.56, 1.04)		
Overall (random-effects model)	659643	53217	1.00 (0.78, 1.27)	48%	0.98	0.79 (0.66, 0.95)	0%	0.01
Overall (fixed-effect model)			0.99 (0.70, 1.38)		0.94	0.79 (0.66, 0.95)		0.01
**Ischaemic stroke**								
MEGASTROKE	440328	34217	1.10 (0.77, 1.57)			0.85 (0.66, 1.10)		
FinnGen	212774	10551	0.80 (0.54, 1.19)			0.83 (0.60, 1.13)		
Overall (random-effects model)	653102	44768	0.95 (0.73, 1.24)	27%	0.73	0.84 (0.69, 1.03)	0%	0.09
Overall (fixed-effect model)			0.95 (0.70, 1.30)		0.75	0.84 (0.69, 1.03)		0.09
**Heart failure**								
HERMES	977323	47309	0.92 (0.66, 1.29)			0.95 (0.75, 1.20)		
FinnGen	195091	23701	0.85 (0.64, 1.12)			0.75 (0.55, 1.03)		
Overall (random-effects model)	1172414	71010	0.88 (0.71, 1.09)	0%	0.23	0.87 (0.72, 1.06)	27%	0.16

Overall (fixed-effect model)			0.88 (0.71, 1.09)		0.23	0.87 (0.69, 1.09)		0.21
**Atrial fibrillation**								
Nielson 2018	1030836	60662	1.09 (0.73, 1.63)			0.98 (0.74, 1.31)		
FinnGen	116926	22068	1.05 (0.61, 1.80)			1.24 (0.90, 1.70)		
Overall (random-effects model)	1147762	82730	1.08 (0.78, 1.48)	0%	0.66	1.09 (0.88, 1.35)	13%	0.42
Overall (fixed-effect model)			1.08 (0.78, 1.48)		0.66	1.09 (0.87, 1.38)		0.45

*: p value for OR.

OR (95%CI) estimates were obtained from the inverse variance weighted methods. OR estimates were for increase of daily one heaped tablespoon of vegetable intake.

Supplementary table S11: Associations between vegetable intake and cardiovascular risk in meta-analysis of univariable Mendelian randomization.

			Cooked vegetable			Raw vegetable		
	Total n	Event n	OR (95%CI)	I2	P*	OR (95%CI)	I2	P*
**Coronary heart diseases**								
CARDIoGRAMplusC4D	184305	60801	1.09 (0.80, 1.48)			0.88 (0.72, 1.08)		
FinnGen	218792	21012	1.07 (0.69, 1.68)			1.06 (0.83, 1.35)		
UK Biobank	361797	37014	1.09 (0.68, 1.74)			0.87 (0.71, 1.06)		
Overall (random-effects model)	764894	118827	1.09 (0.87, 1.36)	0%	0.47	0.92 (0.81, 1.04)	0%	0.18
Overall (fixed-effect model)			1.09 (0.87, 1.36)		0.47	0.92 (0.81, 1.04)		0.18
**Stroke**								
MEGASTROKE	446696	40585	0.99 (0.74, 1.34)			0.91 (0.77, 1.07)		
FinnGen	212947	12632	1.03 (0.62, 1.73)			0.85 (0.65, 1.12)		
UK Biobank	361797	9298	0.59 (0.24, 1.43)			0.92 (0.64, 1.32)		
Overall (random-effects model)	1021440	62515	0.96 (0.75, 1.23)	0%	0.74	0.90 (0.79, 1.02)	0%	0.10
Overall (fixed-effect model)			0.96 (0.75, 1.23)		0.74	0.90 (0.79, 1.02)		0.10
**Ischaemic stroke**								
MEGASTROKE	440328	34217	0.96 (0.68, 1.36)			0.94 (0.79, 1.13)		
FinnGen	212774	10551	0.88 (0.51, 1.53)			0.82 (0.62, 1.08)		
UK Biobank	361797	7264	0.69 (0.25, 1.91)			0.87 (0.58, 1.31)		
Overall (random-effects model)	1014899	52032	0.91 (0.69, 1.21)	0%	0.54	0.90 (0.78, 1.04)	0%	0.14
Overall (fixed-effect model)			0.91 (0.69, 1.21)		0.54	0.90 (0.78, 1.04)		0.14
**Heart failure**								
HERMES	977323	47309	1.38 (1.06, 1.80)			1.09 (0.94, 1.27)		

FinnGen	195091	23701	1.05 (0.75, 1.48)			1.14 (0.93, 1.40)		
UK Biobank	361797	11773	0.77 (0.34, 1.75)			0.83 (0.60, 1.15)		
Overall (random-effects model)	1534211	82783	1.18 (0.90, 1.53)	28%	0.23	1.07 (0.95, 1.20)	27%	0.26
Overall (fixed-effect model)			1.21 (0.99, 1.48)		0.07	1.07 (0.95, 1.20)		0.26
**Atrial fibrillation**								
Nielson 2018	1030836	60662	0.98 (0.79, 1.21)			1.03 (0.83, 1.29)		
FinnGen	116926	22068	1.22 (0.61, 2.47)			1.02 (0.72, 1.46)		
UK Biobank	361797	25915	0.74 (0.42, 1.33)			0.82 (0.66, 1.03)		
Overall (random-effects model)	1509559	108645	0.97 (0.80, 1.17)	0%	0.73	0.94 (0.79, 1.11)	9%	0.46
Overall (fixed-effect model)			0.97 (0.80, 1.17)		0.73	0.94 (0.81, 1.08)		0.38

*: p value for OR.

OR (95%CI) estimates were obtained from the inverse variance weighted methods. OR estimates were for increase of daily one heaped tablespoon of vegetable intake.

Outcome	Total n	Event n	Cooked vegetable OR (95%CI)	Cooked vegetable P value	Cooked vegetable I sqaure	Raw vegetable OR (95%CI)	Raw vegetable P value	Raw vegetable I sqaure
**Coronary heart diseases**								
CARDIoGRAMplusC4D	184305	60801	1.09 (0.80, 1.48)			0.88 (0.72, 1.08)		
FinnGen	218792	21012	1.07 (0.69, 1.68)			1.06 (0.83, 1.35)		
UK Biobank	361797	37014	1.09 (0.68, 1.74)			0.87 (0.71, 1.06)		
**Overall (random-effects model)**	**764894**	**118827**	**1.09 (0.87, 1.36)**	**0.47**	**0%**	**0.92 (0.81, 1.04)**	**0.18**	**0%**
**Overall (fixed-effect model)**			**1.09 (0.87, 1.36)**	**0.47**		**0.92 (0.81, 1.04)**	**0.18**	
**Stroke**								
MEGASTROKE	446696	40585	0.99 (0.74, 1.34)			0.91 (0.77, 1.07)		
FinnGen	212947	12632	1.03 (0.62, 1.73)			0.85 (0.65, 1.12)		
UK Biobank	361797	9298	0.59 (0.24, 1.43)			0.92 (0.64, 1.32)		
**Overall (random-effects model)**	**1021440**	**62515**	**0.96 (0.75, 1.23)**	**0.74**	**0%**	**0.90 (0.79, 1.02)**	**0.10**	**0%**
**Overall (fixed-effect model)**			**0.96 (0.75, 1.23)**	**0.74**		**0.90 (0.79, 1.02)**	**0.10**	
**Ischaemic stroke**								
MEGASTROKE	440328	34217	0.96 (0.68, 1.36)			0.94 (0.79, 1.13)		
FinnGen	212774	10551	0.88 (0.51, 1.53)			0.82 (0.62, 1.08)		
UK Biobank	361797	7264	0.69 (0.25, 1.91)			0.87 (0.58, 1.31)		
**Overall (random-effects model)**	**1014899**	**52032**	**0.91 (0.69, 1.21)**	**0.54**	**0%**	**0.90 (0.78, 1.04)**	**0.14**	**0%**
**Overall (fixed-effect model)**			**0.91 (0.69, 1.21)**	**0.54**		**0.90 (0.78, 1.04)**	**0.14**	
**Heart failure**								
HERMES	977323	47309	1.38 (1.06, 1.80)			1.09 (0.94, 1.27)		
FinnGen	195091	23701	1.05 (0.75, 1.48)			1.14 (0.93, 1.40)		
UK Biobank	361797	11773	0.77 (0.34, 1.75)			0.83 (0.60, 1.15)		
**Overall (random-effects model)**	**1534211**	**82783**	**1.18 (0.90, 1.53)**	**0.23**	**28%**	**1.07 (0.95, 1.20)**	**0.26**	**27%**
**Overall (fixed-effect model)**			**1.21 (0.99, 1.48)**	**0.07**		**1.07 (0.95, 1.20)**	**0.26**	
**Atrial fibrillation**								
Nielson 2018	1030836	60662	0.98 (0.79, 1.21)			1.03 (0.83, 1.29)		
FinnGen	116926	22068	1.22 (0.61, 2.47)			1.02 (0.72, 1.46)		
UK Biobank	361797	25915	0.74 (0.42, 1.33)			0.82 (0.66, 1.03)		
**Overall (random-effects model)**	**1509559**	**108645**	**0.97 (0.80, 1.17)**	**0.73**	**0%**	**0.94 (0.79, 1.11)**	**0.46**	**9%**
**Overall (fixed-effect model)**			**0.97 (0.80, 1.17)**	**0.73**		**0.94 (0.81, 1.08)**	**0.38**	

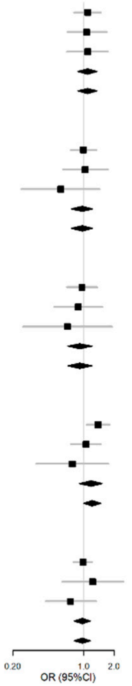

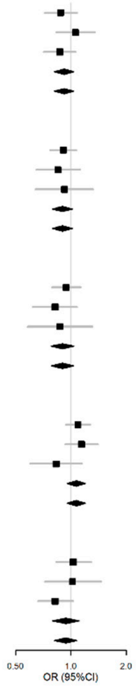

Supplementary figure 4: Associations between vegetable intake and cardiovascular risk in meta-analysis of two- and one-sample univariable Mendelian randomization.

OR (95%CI): odds ratio (95% confidence interval). MR estimates in UK Biobank were obtained from one-sample MR, otherwise two-sample MR.

UKB: UK Biobank. CARDIoGRAMplusC4D: Coronary ARtery DIsease Genome wide Replication and Meta-analysis plus The Coronary Artery Disease Genetics) consortium. MEGASTROKE: MEGASTROKE consortium. HERMES: Heart Failure Molecular Epidemiology for Therapeutic Targets consortium.

Supplementary table S12: The basic characteristics of the SNPs for cooked and raw vegetable intake included in Mendelian randomization analysis for cardiometabolic risk factors

SNP	Chromosome :position	Effect allele	Other allele	Effect allele frequency	Nearest gene	beta	se	p	F statistics
**Cooked vegetable intake**									
rs10020708	4:178097496	C	A	0.51	NEIL3	0.015	0.003	2.52E-06	22.16
rs10156602	9:96345328	G	A	0.36	PHF2	0.020	0.003	4.66E-09	34.33
rs10161952	13:59474383	A	C	0.69	DIAPH3	0.017	0.004	2.60E-06	22.09
rs11995369	8:89649177	C	T	0.20	MMP16	0.023	0.004	2.40E-08	31.14
rs2211345	1:189995554	T	G	0.53	BRINP3	0.016	0.003	1.01E-06	23.91
rs2102738	2:172525884	A	C	0.83	DYNC1I2	0.023	0.004	1.15E-07	28.10
rs442291	2:79676305	C	T	0.39	IL1R1	0.023	0.003	4.03E-12	48.11
rs6420335	13:69556727	C	G	0.53	KLHL1	0.018	0.003	3.07E-08	30.67
rs7521047	1:154076366	T	C	0.54	NUP210L	0.019	0.003	4.75E-09	34.27
**Raw vegetable intake**									
rs1080225	13:59648694	C	G	0.74	DIAPH3	0.024	0.004	4.51E-08	29.92
rs11125813	2:59991047	A	G	0.22	BCL11A	0.023	0.005	7.26E-07	24.55
rs13255011	8:35051793	T	C	0.48	UNC5D	0.021	0.004	6.54E-08	29.20
rs1437761	15:97010698	T	C	0.75	NR2F2	0.023	0.004	1.13E-07	28.13
rs1520919	8:64696606	G	A	0.70	YTHDF3	0.026	0.004	7.55E-10	37.87
rs2447090	17:2298974	A	G	0.64	MNT	0.018	0.004	4.81E-06	20.91
rs2915858	5:166542621	G	A	0.43	TENM2	0.022	0.004	8.80E-09	33.09
rs4845364	1:154141908	G	A	0.49	NUP210L	0.024	0.004	1.91E-10	40.68
rs3828118	1:82443010	A	G	0.70	ADGRL2	0.022	0.004	2.02E-07	26.97
rs6729029	2:176475422	T	C	0.74	LNPK	0.022	0.004	2.12E-07	26.89
rs6079589	20:14850762	C	T	0.78	MACROD2	0.025	0.005	3.09E-08	30.65
rs10973268	9:37254139	C	G	0.54	ZCCHC7	0.021	0.004	8.42E-08	28.80
rs6958768	7:77773693	A	C	0.83	MAGI2	0.026	0.005	6.78E-07	24.68
rs7570469	2:79709354	C	T	0.67	CTNNA2	0.027	0.004	2.70E-11	44.39
rs3120876	13:55746151	C	T	0.06	PRR20A	0.027	0.008	9.35E-04	10.95
rs4838298	9:128629488	C	T	0.64	PBX3	0.028	0.004	1.57E-11	48.82

rs9323534	14:20586432	C	T	0.57	OR4K17	0.022	0.004	2.00E-08	31.49
rs9359954	6:92318594	G	T	0.48	MAP3K7	0.017	0.004	5.93E-06	20.51
rs956362	15:35927655	G	A	0.21	DPH6	0.020	0.005	2.40E-05	17.84

SNP: single nucleotide polymorphism.

Supplementary table S13: Association between vegetable intake and cardiometabolic risk factors in univariable Mendelian randomization

			Inverse-variance weighted method			Weighted median method		MR-Egger method		
	SNP	F statistics	Beta (95%CI)	p	$P_{heterogeneity}$	Beta (95%CI)	p	Beta (95%CI)	p	$P_{intercept}$
TC										
Cooked vegetable	9	30.53	-0.07 (-0.23, 0.08)	0.34	0.96	-0.10 (-0.29, 0.10)	0.34	-0.03 (-1.05, 0.99)	0.96	0.93
Raw vegetable	19	29.28	-0.04 (-0.17, 0.09)	0.54	0.13	-0.11 (-0.27, 0.06)	0.21	0.81 (-0.11, 1.73)	0.08	0.07
TG										
Cooked vegetable	9	30.53	-0.06 (-0.27, 0.15)	0.59	0.03	-0.11 (-0.33, 0.12)	0.35	-0.71 (-2.10, 0.68)	0.31	0.35
Raw vegetable	19	29.28	-0.05 (-0.15, 0.05)	0.33	0.56	0.01 (-0.13, 0.16)	0.84	-0.43 (-1.19, 0.34)	0.27	0.33
HDL										
Cooked vegetable	9	30.53	0.05 (-0.11, 0.22)	0.51	0.31	-0.06 (-0.27, 0.16)	0.60	0.60 (-0.45, 1.66)	0.26	0.30
Raw vegetable	19	29.28	0.04 (-0.06, 0.15)	0.42	0.44	0.05 (-0.10, 0.20)	0.50	0.08 (-0.73, 0.89)	0.85	0.93
LDL										
Cooked vegetable	9	30.53	-0.02 (-0.18, 0.14)	0.79	0.84	-0.02 (-0.23, 0.18)	0.83	-0.26 (-1.30, 0.79)	0.63	0.66
Raw vegetable	19	29.28	-0.04 (-0.20, 0.11)	0.58	0.01	-0.06 (-0.23, 0.11)	0.50	0.71 (-0.43, 1.85)	0.22	0.19
BMI										
Cooked vegetable	9	30.53	-0.12 (-0.27, 0.02)	0.09	0.21	-0.20 (-0.37, -0.03)	0.02	-0.56 (-1.53, 0.42)	0.26	0.38
Raw vegetable	19	29.28	0.05 (-0.05, 0.16)	0.33	0.04	-0.01 (-0.12, 0.11)	0.91	-0.98 (-1.66, -0.30)	< 0.01	< 0.01*
OGTT										
Cooked vegetable	9	30.53	0.04 (-0.26, 0.34)	0.77	0.57	0.09 (-0.32, 0.50)	0.67	-0.75 (-2.75, 1.25)	0.46	0.43

Raw vegetable	19	29.28	-0.23 (-0.42, -0.04)	0.02	0.70	-0.29 (-0.56, -0.02)	0.03	-0.74 (-2.24, 0.76)	0.33	0.50
FG										
Cooked vegetable	9	30.53	0.01 (-0.07, 0.09)	0.85	0.09	0.02 (-0.07, 0.11)	0.69	-0.32 (-0.86, 0.22)	0.24	0.23
Raw vegetable	19	29.28	0.04 (-0.02, 0.09)	0.18	0.02	0.04 (-0.02, 0.10)	0.22	0.23 (-0.20, 0.65)	0.29	0.38
FI										
Cooked vegetable	9	30.53	0.03 (-0.04, 0.11)	0.36	0.47	0.02 (-0.07, 0.12)	0.61	-0.27 (-0.75, 0.21)	0.27	0.21
Raw vegetable	19	29.28	0.01 (-0.04, 0.06)	0.79	0.18	0.01 (-0.05, 0.08)	0.74	0.46 (0.11, 0.82)	0.01	0.01**
HbA1c										
Cooked vegetable	9	30.53	-0.02 (-0.07, 0.03)	0.43	0.85	-0.01 (-0.08, 0.05)	0.64	0.09 (-0.23, 0.42)	0.57	0.49
Raw vegetable	19	29.28	-0.02 (-0.05, 0.00)	0.09	0.50	-0.04 (-0.08, 0.01)	0.10	-0.14 (-0.37, 0.10)	0.25	0.34
SBP										
Cooked vegetable	9	30.53	-1.05 (-4.31, 2.20)	0.53	0.04	-1.67 (-5.05, 1.71)	0.33	-4.90 (-28.17, 18.38)	0.68	0.74
Raw vegetable	19	29.28	-0.39 (-2.08, 1.30)	0.65	0.10	-1.48 (-3.53, 0.58)	0.16	3.93 (-9.71, 17.58)	0.57	0.53
DBP										
Cooked vegetable	9	30.53	-0.89 (-2.73, 0.95)	0.34	0.10	-0.16 (-2.30, 1.99)	0.89	0.13 (-13.10, 13.37)	0.98	0.88
Raw vegetable	19	29.28	-0.50 (-1.54, 0.54)	0.34	0.10	-0.29 (-1.56, 0.97)	0.65	-1.98 (-10.42, 6.46)	0.65	0.73
PP										
Cooked vegetable	9	30.53	-0.58 (-3.09, 1.93)	0.65	0.01	-0.89 (-3.35, 1.57)	0.48	-6.81 (-24.27, 10.65)	0.44	0.48
Raw vegetable	19	29.28	-0.18 (-1.28, 0.92)	0.75	0.14	-0.69 (-2.05, 0.67)	0.32	4.26 (-4.44, 12.96)	0.34	0.31

SNP: single nucleotide polymorphism. TC: total cholesterol. TG: triglyceride. HDL: high density lipoprotein. LDL: low density lipoprotein. BMI: body mass index. OGTT: 2-hour glucose after oral glucose tolerance test. FG: fasting glucose. FI: fasting insulin. HbA1c: glycated haemoglobin. SBP: systolic blood pressure. DBP: diastolic blood pressure. PP: pulse pressure.

*BMI: P for MR-Egger intercept < 0.05, MR-PRESSO method was performed and identified 1 outlier SNP (rs6729029); the MR-PRESSO corrected estimate was 0.02 (-0.07, 0.11), p for distortion test 0.65.

**FI: P for MR-Egger intercept < 0.05, MR-PRESSO method was performed and identified 0 outlier SNPs; the MR-PRESSO corrected estimate remained 0.01 (-0.04, 0.06).

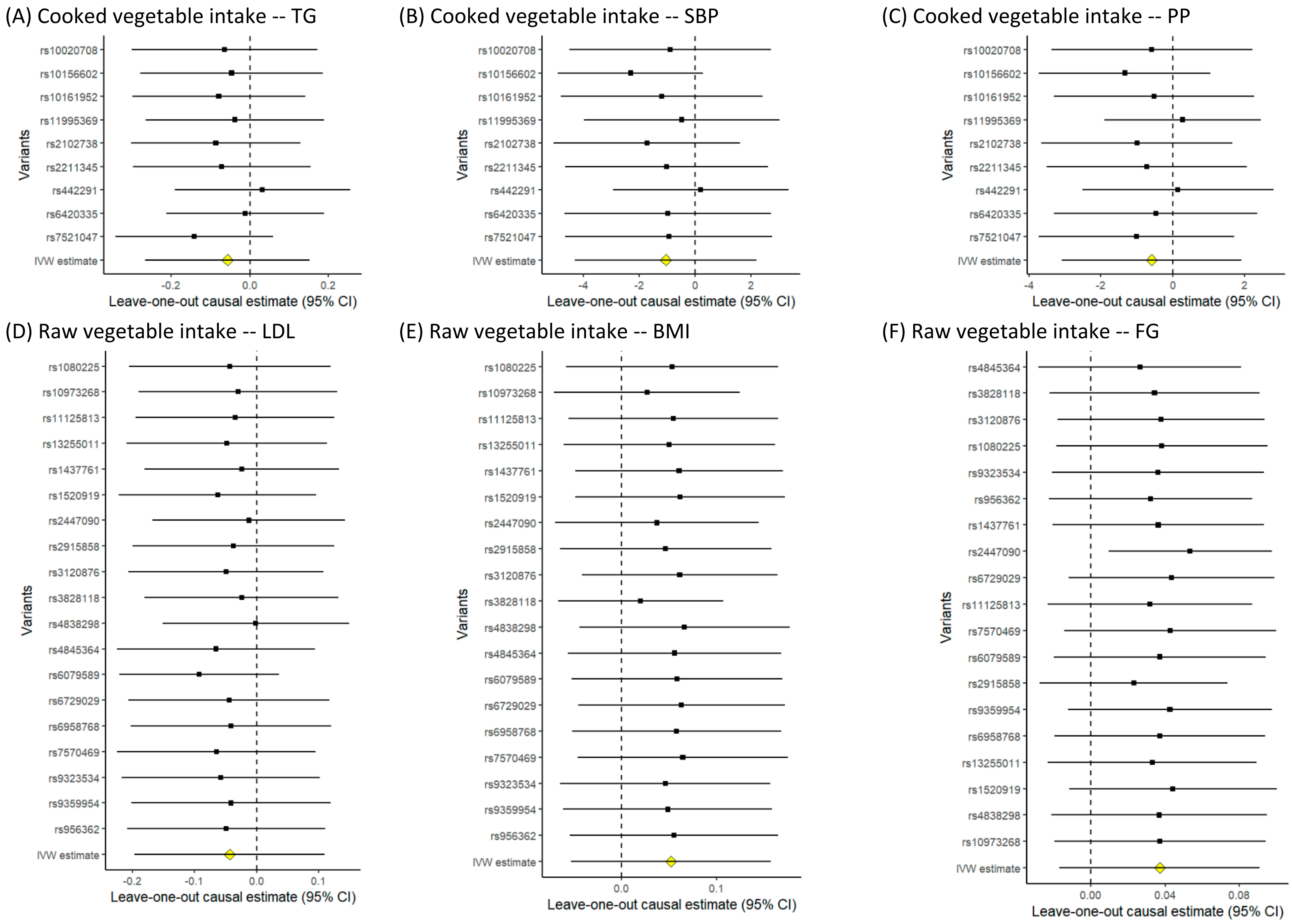
(A) Cooked vegetable intake -- TG
Variants
rs10020708
rs10156602
rs10161952
rs11995369
rs2102738
rs2211345
rs442291
rs6420335
rs7521047
IVW estimate
-0.2
0.0
0.2
Leave-one-out causal estimate (95% CI)
(B) Cooked vegetable intake -- SBP
Variants
rs10020708
rs10156602
rs10161952
rs11995369
rs2102738
rs2211345
rs442291
rs6420335
rs7521047
IVW estimate
-4
-2
0
2
Leave-one-out causal estimate (95% CI)
(C) Cooked vegetable intake -- PP
Variants
rs10020708
rs10156602
rs10161952
rs11995369
rs2102738
rs2211345
rs442291
rs6420335
rs7521047
IVW estimate
-4
-2
0
2
Leave-one-out causal estimate (95% CI)
(D) Raw vegetable intake -- LDL
Variants
rs1080225
rs10973268
rs11125813
rs13255011
rs1437761
rs1520919
rs2447090
rs2915858
rs3120876
rs3828118
rs4838298
rs4845364
rs6079589
rs6729029
rs6958768
rs7570469
rs9323534
rs9359954
rs956362
IVW estimate
-0.2
-0.1
0.0
0.1
Leave-one-out causal estimate (95% CI)
(E) Raw vegetable intake -- BMI
Variants
rs1080225
rs10973268
rs11125813
rs13255011
rs1437761
rs1520919
rs2447090
rs2915858
rs3120876
rs3828118
rs4838298
rs4845364
rs6079589
rs6729029
rs6958768
rs7570469
rs9323534
rs9359954
rs956362
IVW estimate
0.0
0.1
Leave-one-out causal estimate (95% CI)
(F) Raw vegetable intake -- FG
Variants
rs4845364
rs3828118
rs3120876
rs1080225
rs9323534
rs956362
rs1437761
rs2447090
rs6729029
rs11125813
rs7570469
rs6079589
rs2915858
rs9359954
rs6958768
rs13255011
rs1520919
rs4838298
rs10973268
IVW estimate
0.00
0.04
0.08
Leave-one-out causal estimate (95% CI)

Supplementary figure S5: Forest plots for the leave-one-out analysis for selected genetic associations between vegetable intake and cardiometabolic risk factors.

TG: triglyceride. LDL: low density lipoprotein. BMI: body mass index. FG: fasting glucose. FI: fasting insulin. SBP: systolic blood pressure. PP: pulse pressure. Removing the SNP (rs2447090) for raw vegetable intake-FG association, the effect was 0.05 (0.01, 0.10; p = 0.02), not passing the Bonferroni corrected significance level (0.002).

Supplementary table S14: Association between vegetable intake and body mass index, serum lipids in multivariable Mendelian randomization

	Inverse-variance weighted method			MR-Egger method			Median Method	
	Beta (95%CI)	p	$P_{heterogeneity}$	Beta (95%CI)	p	$P_{intercept}$	Beta (95%CI)	p
TC								
Cooked vegetable	-0.06 (-0.30, 0.18)	0.62	0.40	-0.01 (-0.35, 0.33)	0.96	0.68	0.01 (-0.31, 0.33)	0.96
Raw vegetable	-0.02 (-0.20, 0.16)	0.84		0.03 (-0.26, 0.31)	0.85		-0.11 (-0.36, 0.14)	0.41
TG								
Cooked vegetable	-0.12 (-0.36, 0.13)	0.35	0.17	-0.16 (-0.51, 0.18)	0.36	0.71	-0.25 (-0.56, 0.07)	0.13
Raw vegetable	0.03 (-0.15, 0.22)	0.71		-0.01 (-0.30, 0.28)	0.95		0.16 (-0.07, 0.39)	0.17
HDL								
Cooked vegetable	0.07 (-0.16, 0.29)	0.57	0.43	0.26 (-0.05, 0.57)	0.1	0.08	-0.14 (-0.46, 0.19)	0.41
Raw vegetable	0.02 (-0.15, 0.18)	0.84		0.20 (-0.06, 0.46)	0.14		0.12 (-0.12, 0.35)	0.33
LDL								
Cooked vegetable	0.00 (-0.29, 0.29)	0.99	0.06	0.07 (-0.34, 0.48)	0.74	0.65	0.06 (-0.30, 0.42)	0.74
Raw vegetable	-0.04 (-0.26, 0.18)	0.72		0.02 (-0.32, 0.36)	0.9		-0.06 (-0.35, 0.22)	0.66
BMI								
Cooked vegetable	-0.18 (-0.41, 0.05)	0.13	0.02	-0.31 (-0.63, 0.01)	0.06	0.25	-0.21 (-0.47, 0.05)	0.12
Raw vegetable	0.12 (-0.05, 0.29)	0.15		-0.00 (-0.28, 0.27)	0.98		0.09 (-0.11, 0.28)	0.38
OGTT								
Cooked vegetable	0.22 (-0.20, 0.64)	0.31	0.75	-0.06 (-0.66, 0.54)	0.85	0.20	0.29 (-0.27, 0.85)	0.31

Raw vegetable	-0.33 (-0.64, -0.01)	0.04		-0.59 (-1.11, -0.08)	0.02		-0.27 (-0.68, 0.15)	0.21
FG								
Cooked vegetable	0.01 (-0.11, 0.13)	0.84	0.01*	0.06 (-0.11, 0.23)	0.52	0.48	0.01 (-0.11, 0.14)	0.85
Raw vegetable	0.03 (-0.06, 0.11)	0.56		0.07 (-0.08, 0.21)	0.36		0.03 (-0.06, 0.12)	0.49
FI								
Cooked vegetable	0.02 (-0.09, 0.13)	0.71	0.21	0.04 (-0.12, 0.20)	0.62	0.74	0.01 (-0.14, 0.15)	0.90
Raw vegetable	0.00 (-0.08, 0.08)	0.99		0.02 (-0.12, 0.16)	0.79		0.01 (-0.09, 0.12)	0.79
HbA1c								
Cooked vegetable	-0.01 (-0.08, 0.06)	0.74	0.73	-0.02 (-0.11, 0.07)	0.66	0.76	0.00 (-0.08, 0.09)	0.98
Raw vegetable	-0.02 (-0.07, 0.03)	0.47		-0.03 (-0.11, 0.05)	0.5		-0.02 (-0.08, 0.04)	0.51
SBP								
Cooked vegetable	-2.43 (-6.36, 1.49)	0.23	0.04	-3.70 (-9.41, 2.00)	0.21	0.55	-0.76 (-5.49, 3.97)	0.75
Raw vegetable	0.94 (-1.91, 3.78)	0.52		-0.31 (-5.24, 4.63)	0.9		-0.97 (-4.31, 2.38)	0.57
DBP								
Cooked vegetable	-1.04 (-3.38, 1.29)	0.38	0.07	-3.15 (-6.37, 0.07)	0.06	0.07	-1.95 (-4.99, 1.09)	0.21
Raw vegetable	-0.12 (-1.81, 1.57)	0.89		-2.18 (-4.97, 0.60)	0.13		0.72 (-1.39, 2.84)	0.51
PP								
Cooked vegetable	-1.56 (-4.32, 1.20)	0.27	0.02	-1.00 (-5.02, 3.02)	0.63	0.71	-1.12 (-4.76, 2.51)	0.55
Raw vegetable	0.82 (-1.18, 2.81)	0.42		1.37 (-2.11, 4.85)	0.44		-0.14 (-2.62, 2.35)	0.91

TC: total cholesterol. TG: triglyceride. HDL: high density lipoprotein. LDL: low density lipoprotein. BMI: body mass index. OGTT: 2-hour glucose after oral glucose tolerance test. FG: fasting glucose. FI: fasting insulin. HbA1c: glycated haemoglobin. SBP: systolic blood pressure. DBP: diastolic blood pressure. PP: pulse pressure.

*: removing the potential outlier SNP rs2447090 yielded -0.01 (-0.10, 0.10; p = 0.97) and 0.05 (-0.03, 0.12; p = 0.23) for cooked and raw vegetable intake, respectively.
